# Variant Callers for Next-Generation Sequencing Data: A Comparison Study

**DOI:** 10.1371/journal.pone.0075619

**Published:** 2013-09-27

**Authors:** Xiangtao Liu, Shizhong Han, Zuoheng Wang, Joel Gelernter, Bao-Zhu Yang

**Affiliations:** 1 Department of Psychiatry, Division of Human Genetics, Yale University School of Medicine, New Haven, Connecticut, United States of America; 2 VA CT Health Care Center, West Haven, Connecticut, United States of America; 3 Department of Biostatistics, Yale School of Public Health, New Haven, Connecticut, United States of America; 4 Departments of Genetics and Neurobiology, Yale University School of Medicine, New Haven, Connecticut, United States of America; Memorial Sloan Kettering Cancer Center, United States of America

## Abstract

Next generation sequencing (NGS) has been leading the genetic study of human disease into an era of unprecedented productivity. Many bioinformatics pipelines have been developed to call variants from NGS data. The performance of these pipelines depends crucially on the variant caller used and on the calling strategies implemented. We studied the performance of four prevailing callers, SAMtools, GATK, glftools and Atlas2, using single-sample and multiple-sample variant-calling strategies. Using the same aligner, BWA, we built four single-sample and three multiple-sample calling pipelines and applied the pipelines to whole exome sequencing data taken from 20 individuals. We obtained genotypes generated by Illumina Infinium HumanExome v1.1 Beadchip for validation analysis and then used Sanger sequencing as a “gold-standard” method to resolve discrepancies for selected regions of high discordance. Finally, we compared the sensitivity of three of the single-sample calling pipelines using known simulated whole genome sequence data as a gold standard. Overall, for single-sample calling, the called variants were highly consistent across callers and the pairwise overlapping rate was about 0.9. Compared with other callers, GATK had the highest rediscovery rate (0.9969) and specificity (0.99996), and the Ti/Tv ratio out of GATK was closest to the expected value of 3.02. Multiple-sample calling increased the sensitivity. Results from the simulated data suggested that GATK outperformed SAMtools and glfSingle in sensitivity, especially for low coverage data. Further, for the selected discrepant regions evaluated by Sanger sequencing, variant genotypes called by exome sequencing versus the exome array were more accurate, although the average variant sensitivity and overall genotype consistency rate were as high as 95.87% and 99.82%, respectively. In conclusion, GATK showed several advantages over other variant callers for general purpose NGS analyses. The GATK pipelines we developed perform very well.

## Introduction

Next-generation sequencing technology has produced a gigantic amount of biological data and has shed light on the path towards personalized medicine. While the cost of high throughput genome sequencing is decreasing in terms of merely acquiring sequence data, the analysis and interpretation of these large-scale sequencing data continues to pose a major challenge [1]. To call variants from this NGS data, many aligners and variant callers have been developed and composed into diverse pipelines. A typical pipeline contains an aligner and a variant caller: the aligner maps the sequencing reads to a reference genome, and the variant caller identifies variant sites and assigns a genotype to the subject(s). The performances of different aligners have been studied extensively [1-3]. Overall, Burrows-Wheeler Transform (BWT)-based aligners are fast and memory efficient, because alignment is a string matching process and BWT-based aligners employ data compression features by creating an index of the reference genome to facilitate string matching. The Burrows-Wheeler Aligner (BWA) [4] displays a good balance between running time, memory usage, and accuracy [5]. For a systematic comparison, we chose BWA as the common aligner for all the variant callers and calling strategies which we would implement.

Variant calling consists of two steps: genotype assignment and variant identification. Early probabilistic methods, such as Mapping and Assembly with Quality (MAQ) [6] and SOAPsnp [7], used fixed prior values for heterozygote probabilities and nucleotide-read error probabilities. SeqEM [8] introduces multiple-sample genotype calling via an adaptive approach employing the expectation-maximization (EM) algorithm to estimate the model parameters. Hardy-Weinberg equilibrium (HWE) could optionally be assumed so that there would be fewer parameters for EM algorithm to estimate.

The most widely used variant callers include SAMtools [9], glftools [10], GATK [11,12], and Atlas2 [13]. Table 1 presents simple summaries of their characteristics. SAMtools uses a revised MAQ model to estimate sequencing error. The glftools family (glfSingle, glfMultiples, and polymutt) call SNPs from pre-generated genotype likelihood files (GLF). GATK adopts the MapReduce philosophy [14] to parallel programming for simple Bayesian modeling. Atlas2 employs logistic regression models trained on validated whole-exome capture sequencing data rather than regular likelihood calculations and has been shown to have high sensitivity [15]. All of these variant callers have been widely used for previous NGS analyses such as the 1000 Genomes Project [16]. Additionally, they can be flexibly implemented as part of a customized pipeline. However, the performances of these callers with different calling strategies have not been previously compared in a deliberate and systematic way.

**Table 1:** Summaries of the variant-callers.

**Caller**	**SAMtools**	**GATK**	**glfTools**	**Atlas2**
Code	C	Java	C & C++	Ruby
Model	HMM & MAQ	Bayesian	Likelihood-based	Logistic regression
Algorithm	EM	MapReduce	-	-
Sampling	Single & multiple	Single & multiple	Single & multiple	Single
Variants	SNPs & indels	SNPs & indels	SNPs only	SNPs & indels
Features	Sorting, indexing, formatting, etc.	Realignment, per base recalibration, VQSR	Requires pre-generated GLFs generated by SAMtools-hybrid	Separated programs for SNPs and indels

To that end, we built several pipelines with the same pre-calling procedure before variant calling and applied the pipelines to real exome sequencing data and simulated whole genome sequencing (WGS) data. Statistical metrics were reported to evaluate the callers and calling strategies. The aim was to provide a comprehensive evaluation for the most widely used variant callers and strategies and to assist researchers in choosing a suitable variant caller and strategy for their own NGS studies.

## Methods

### The pipelines

**Figure 1:**
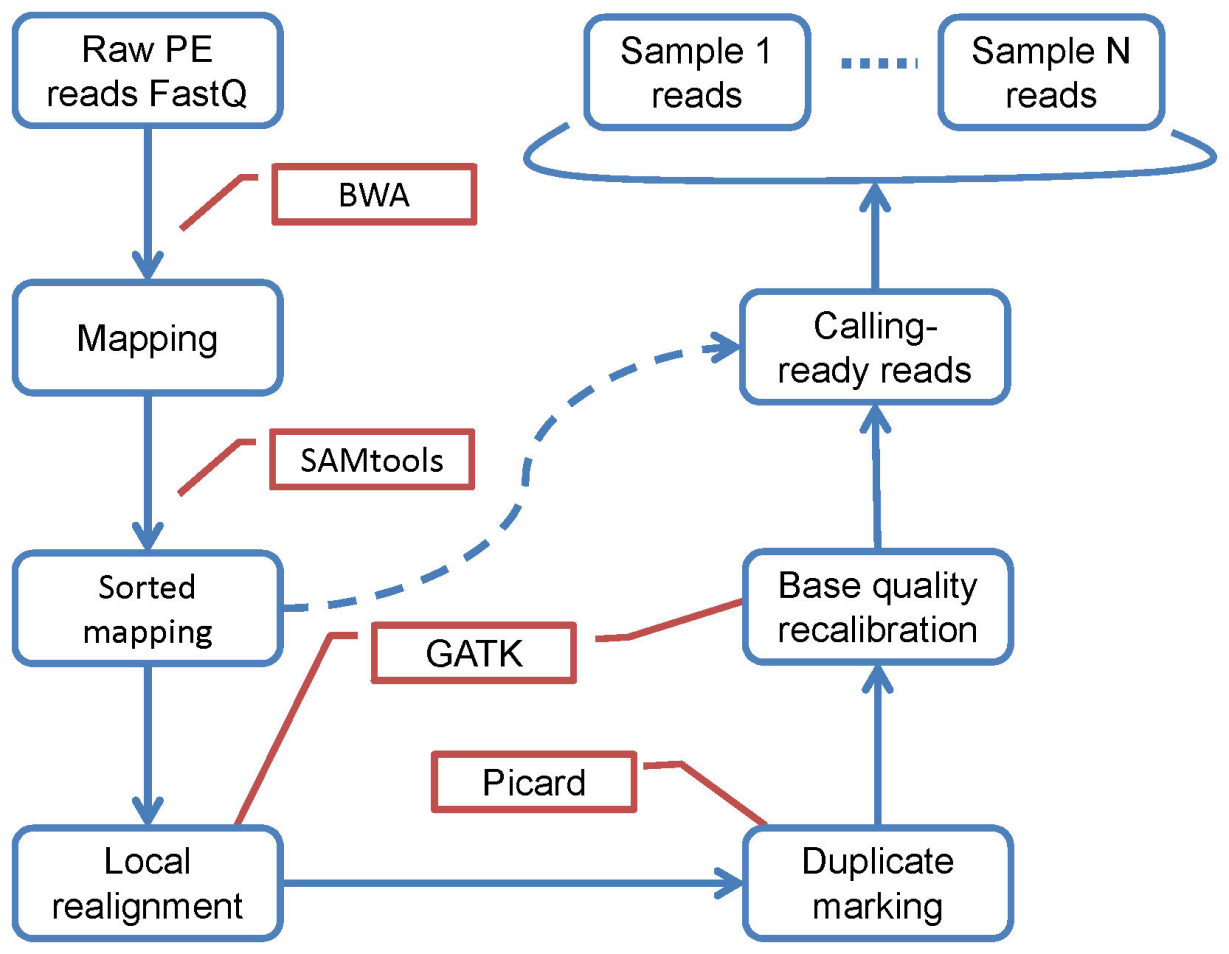
Unified steps of the pipelines. Blue rounded rectangles represent the reads, blue rectangles represent mapping-QC procedures, red callouts indicate the tools. The dashed curve arrow represents a reduced version skipping the mapping-QC steps.

Our pipelines were customized for systematic evaluation. For that purpose, the alignment and several mapping-improvement steps were unified for all compared variant callers as shown in Figure 1. The paired-end reads in FastQ format were mapped to reference genome HG19 using BWA-0.6.1. The mapping files in SAM (Sequence Alignment/Map) format were converted to BAM (binary version of SAM) format and sorted by SAMtools-0.1.18. Local realignment around known indels was performed by GATK-1.6.9 on the sorted BAM files. Picard-tools-1.5.3 [17] was used to remove PCR duplicates. Finally, base quality score recalibration was performed using GATK again. These steps constitute a unified procedure generating BAM files ready for variant-calling. A reduced version skips the mapping QC steps, as marked by the dashed curve arrow in Figure 1. Hereafter, we use “Unified” and “Reduced” to indicate the two versions of the pre-calling procedure. We built four single-sample calling pipelines and three multiple-sample calling pipelines as follows:

1 Unified pre-calling procedure + SAMtools single-sample calling;
2 Unified pre-calling procedure + SAMtools multiple-sample calling;
3 Unified pre-calling procedure + GATK single-sample calling;
4 Unified pre-calling procedure + GATK multiple-sample calling;
5 Unified pre-calling procedure + SAMtools-0.1.7a-hybrid + glfSingle;
6 Unified pre-calling procedure + SAMtools-0.1.7a-hybrid + glfMultiples
7 Unified pre-calling procedure + Atlas2 v1.0.

For all seven pipelines, the “Unified” pre-calling procedure can be replaced by the “Reduced” version.

### Whole exome sequence data

We sequenced exomes of 20 individuals. Twelve of them were from a single pedigree, while the other eight were unrelated individuals. Participants recruited early in the study were each given an "Information About" sheet, and consent was oral and documented in an anonymized form. Participants recruited later in the study provided written, informed consent. Both consent procedures, and these studies, were approved by the Yale IRB. The sequencing method allocated these individuals into five lanes and used four bar codes such that each individual had a unique sequencing ID. Nimblegen EZ Exome V2 capture was used and contained 244,619 intervals covering about 44.1 megabases. We used the Illumina HiSeq platform for sequencing and generated 74 bp paired-end reads at the Yale Center for Genome Analysis.

We first ran FastQC [18] to summarize the reads (data not shown) with a focus on base quality scores and GC content, N content, sequence duplication levels. The unified pre-calling procedure described above was applied to the reads of all 20 individuals, and mapping files ready for variant-calling were generated. In local realignment, we used a BED (Browser Extensible Data) file downloaded from the UCSC genome browser to define predetermined exome target regions. This BED file contained all known exons in RefGene plus 10 bases on both ends of each interval, extended in aim to cover more total bases on human genome than the Nimblegen EZ Exome V2 capture regions. To evaluate the mapping by BWA, we used the SAMtools “idxstats” command to count the numbers of mapped and unmapped reads and GATK “DepthOfCoverage” to compute the read depths at bases in target regions in the BED file. The distinct variant-calling procedures of the pipelines generated raw variants from the final BAM files with specific command option settings. The raw variants were further filtered; for example, VCFtools-0.1.7 [19] was used to filter the variants with the BED file. Finally, we used ANNOVAR [20] to perform regional and functional annotations. Details about the pipeline implementation can be found in the first section of File S1. The numbers of SNPs and indels and transition/transversion (Ti/Tv) ratios were used as metrics to evaluate callers, calling strategies, and filters. In addition, we checked the overlapping between sets of filtered variants by the four single-sample calling pipelines.

### Exome array data

To validate the variants, we also generated microarray data using the Illumina HumanExome v1.1 Beadchip [21] for 12 out of those 20 individuals: six from the pedigree, and six from the eight unrelated individuals. The Illumina exome chip contains selected non-synonymous, splicing, and stop altering variants, randomly selected synonymous SNPs, GWAS tag SNPs, Ancestry Informative Markers (AIMs), fingerprint SNPs, HLA tag SNPs, mitochondrial SNPs, Chromosome Y SNPs, and over 100 indels, etc.

We used a set of Perl programs to perform quality control on the exome genotype data and compared the exome genotypes with the genotypes of the filtered variant sets by the sequencing pipelines. To compare the rediscovery rate, sensitivity, and specificity of the pipelines, we defined true positives (TPs), false positives (FPs), true negatives (TNs), and false negatives (FNs) in a way similar to binary classification (see Table S1 in File S1 and the exome array data processing section in the supplementary materials). The rediscovery rate, defined as the proportion of called variants at matched sites confirmed by the exome array genotype data, was used as the key metric for evaluating the performance of the variant caller and calling strategy.

### Resolving Discrepancies by Sanger sequencing

We conducted another validation study by Sanger sequencing for a panel of selected variants, with particularly high discordance between the genotypes called from exome sequencing and the exome array for the twelve individuals with both exome sequencing and exome array data. We first chose six exonic variants obtained via the GATK multiple-sample calling pipeline with discordance rate at least 8/12. In other words, for each variant, at most four out of the twelve successfully genotyped individuals had matched genotypes from exome sequencing and the exome array. Then we extended from the selected variants in both 5’ and 3’ directions to target regions of about 400 bps for Sanger sequencing which in each case covered the exon start position. The PCR products generated for sequencing also included a total of seven other nearby variants. Both the discordant variants, and the additional variants that mapped close to them within the targeted amplicons, were evaluated by Sanger sequencing. Non-variant sites with all 0/0 genotypes in the arrays were excluded (as they were completely concordant).

### Simulation of whole genome sequence data

Whole genome sequence data was simulated for two purposes: to provide a “gold standard” (that is, because the exact actual sequence we generated was known) to compare the performances of SAMtools, GATK and glftools on whole genome data; and to check the effect of coverage on variant-calling. Atlas2 was designed for exome sequence analysis [13], so it was excluded for this comparison.

The whole genome sequence data was generated using dwgsim-0.1.10 [22] directly from chromosome 22 of reference genome HG19. Chromosome 22 was chosen for convenience for illustration since it is the smallest autosome. Five independent mutation sets (individuals) were simulated using default parameter settings, which generated 70bp paired-end reads for the five individuals with different average coverage at 4x, 10x, 20x, 40x, 100x (see the simulation and analysis section in the supplementary materials).

We replaced the “Unified” pre-calling procedure in the first three pipelines with “Reduced” and applied them to the simulated WGS data:

1 Reduced pre-calling procedure + SAMtools single-sample calling;
2 Reduced pre-calling procedure + GATK single-sample calling;
3 Reduced pre-calling procedure + SAMtools-0.1.7a-hybrid + glfSingle.

We validated the called variants against the mutant variants of the five “individuals” using VCFtools-0.1.7, which directly provided TPs, FPs, and FNs. The positive prediction value (PPV, the fraction of validated variants among all called from the sequence data) and sensitivity (the fraction of simulated variants which were called from the sequence data) were used as metrics for evaluating the callers. The sensitivity was the key measure.

Data cannot be released due to patient privacy concerns, specifically the identifying nature of the pedigree. Researchers who wish to reanalyze or reproduce this analysis will be granted in-house access to the raw data upon request.

## Results

### Real exome sequence and array data

To evaluate the “Unified” pre-calling procedures of all pipelines, we extracted the number of reads for each individual and computed the percentage of mapped reads, the percentage of mapped unique reads, the number of bases in targeted regions covered by at least one read, and the mean coverage depth (see Table S2 in File S1). Overall, 95.9±1.0% of the reads was mapped to the reference genome (HG19), and 90.7±1.7% of the reads was mapped as unique reads. These show that BWA makes use of a high proportion of the read data. Within the extended exome regions, 42.6-47.8 megabases (57.3-64.3%) were covered by at least 1x, with average coverage ranging from 62x to 137x. On average, 37.33±0.82 megabases (47.3-52.4%) were covered by at least 10x, and 35.59±0.97 megabases (44.1-49.7%) were covered by at least 15x.

**Figure 2:**
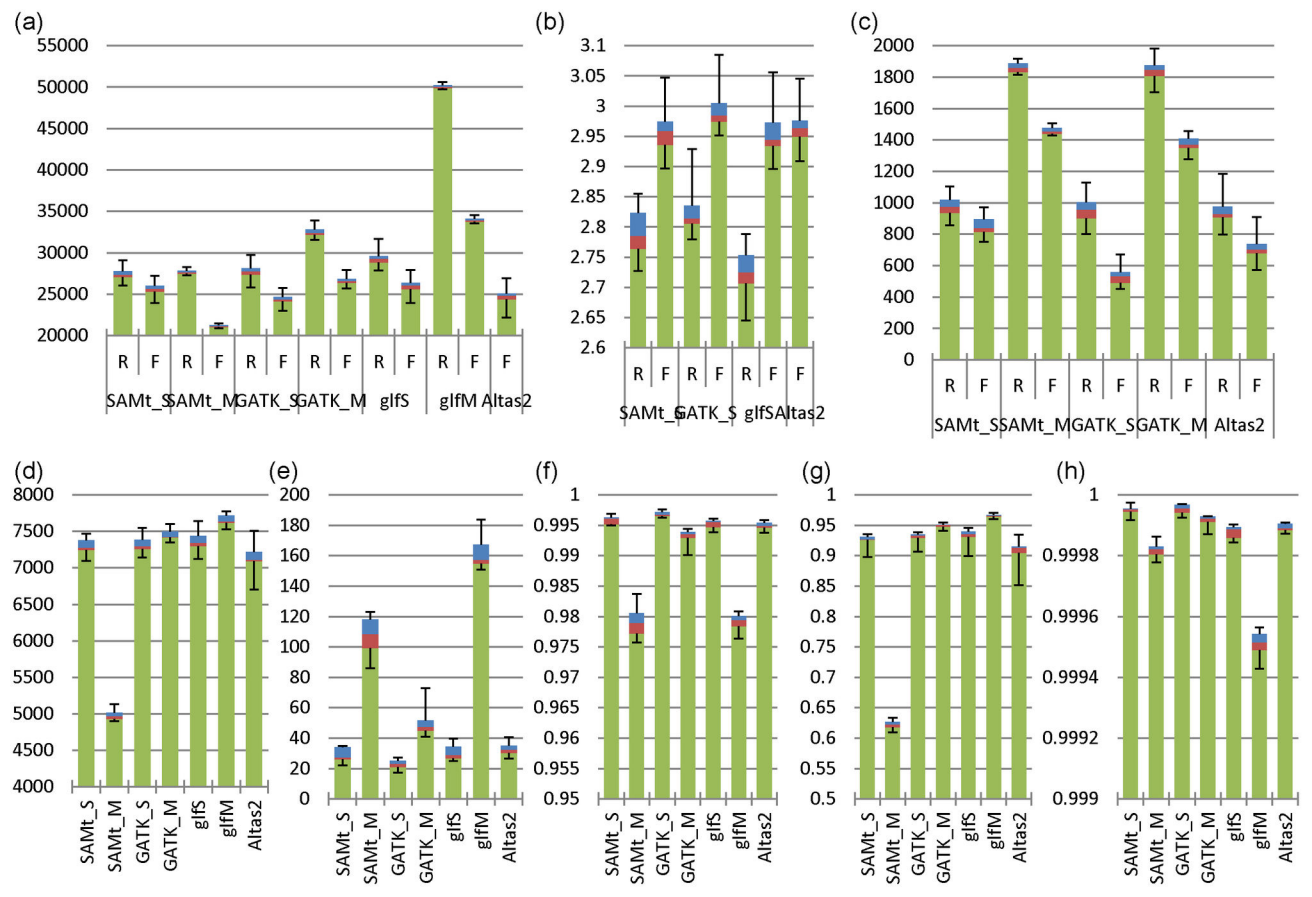
Boxplots of measure for validation by the pipelines. a. Number of SNPs. b. Ti/Tv ratio. c. Number of indels by the pipelines. d. True positive calls by the pipelines. e. False positive calls plus error genotypes by the pipelines. f. Re-discovery rate (positive prediction value) by the pipelines. g. Sensitivities of the pipelines. h. Specificities of the pipelines. The green bars indicate the first quartiles, red bars extend to medians, blue bars reach the third quartile, and error bar caps show the ranges. SAMt, glfS and glfM stand for SAMtools, glfSingle and glfMultiples respectively. “_S” and “_M” represent single and multiple calling strategies. R and F represent raw and filtered variants.

We first compared the numbers of SNPs and indels, and the Ti/Tv ratio, across pipelines. Figure 2a summarizes the distribution of the number of SNPs called from the 20 individuals using different callers and calling strategies (see Figures S1-S3 and the single-sample calling and multiple-sample calling subsections in the supplementary materials (File S1) for individual results). The single-sample calling strategy generated 27.45±0.64k, 27.92±0.85k and 29.35±0.79k raw SNPs with SAMtools, GATK, and glfSingle, respectively; variant filtering removed 6.44%, 12.53%, 11.12% of raw SNPs respectively. These numbers are consistent with the 20.28±0.64k for average observed coding region variants in European American (see Table 1 in [23]), since we used extended target regions described above. In contrast, the multiple-sample calling strategy increased the numbers of called raw SNPs by 0.87%, 16.56% and 70.96% with SAMtools, GATK, and glftools, respectively; variant filtering removed 18.18%, 11.1% and 32.16% of raw variants. Atlas-SNP2 called 24.80±0.98k SNPs after filtering. Notably, compared with single-sample calling after variants filtering, while the GATK and glftools increased the proportion of called variants, the SAMtools multiple-sample calling lost (i.e., did not identify)17.60% SNPs. This might indicate that multiple-sample calling raises the sensitivities of GATK and glftools, but fails on SAMtools most likely due to the default depth limit of SAMtools. Figure 2b summarizes the Ti/Tv ratio of the SNP sets by four single-sample calling pipelines. The raw SNPs have average Ti/Tv ratio 2.79, 2.79, and 2.73 for SAMtools, GATK, and glfSingle, while the filtered have 2.96, 2.99, 2.96 and 2.97 (Atlas2), closer to the expected value 3.02 (see Table S2 in [24]). These confirmed that filtering is important to improve variant calling quality. Figure 2c summarizes the number of indels by SAMtools, GATK and Atlas2. There were no systematic differences for raw indels, but remarkable differences after filtering, which suggested that filters applied to GATK indels and filtering on the posterior probability for Atlas2 indels are more stringent than filtering on variant quality score.

We also compared the numbers of true positives (TPs), false positives (FPs), rediscovery rate, sensitivity and specificity of the pipelines (see Figures S4 and S5, and the exome array results section in the supplementary materials (File S1) for individual results). At the 246,305 unique exome array sites, the 12 individuals on average had 228,816.17±135.75 homozygous reference genotypes, 10,370.92±190.86 heterozygous genotypes, 6,903.08±116.17 homozygous alternate genotypes, and 214.83±148.18 missing genotypes. Table 2 presents genotype summaries for each individual as well as the sequencing coverage on these sites extracted from the corresponding ready-to-call BAM files. Figure 2d and 2e show the summaries of the numbers of TPs and FPs (including error genotypes) from exome array validation, respectively. Six pipelines performed comparably regarding the number of TPs, with each identifying over 7,000 on average. The exception was pipeline 2 (SAMtools multiple-sample calling), which failed to detect enough TPs (4,979.75±66.05). More precisely, GATK generated more TPs than SAMtools and Altas2, and glftools generated the most TPs with single or multiple. GATK (single) produced the least FPs (22.75±3.4) and glfMultiples generated the highest number of FPs. Figure 2f-2h show the rediscovery rate, sensitivity and specificity of the pipelines. Although all pipelines had specificity higher than 0.999, GATK had slight advantages in both rediscovery rate and specificity over other callers. Glftools, especially glfMultiples, had the highest sensitivity, but produced the most FPs at the same time, mostly due to the genotype assignments to those individuals without enough read depth at the array marker sites. GATK multiple-sample calling reduced such inaccurate assignments by placing missing genotypes, and conduced to average specificity 0.999937, which was accompanied by average sensitivity 95.87% (see the 4^th^ bar in Figure 2g).

**Table 2:** Summaries of exome array data by individual and the exome sequencing coverage depths on the array positions in target regions.

**Subject**	**Genotype**				**Coverage**		
	**0/0**	**0/1**	**1/1**	**missing**	**0x**	**>=1x**	**>=20x**
1	228882	10219	7036	168	2178	228877	206196
2	228712	10488	6952	153	1510	229545	219000
4	229058	10177	6868	202	2254	228801	205724
5	228725	10370	6939	270	2091	228964	207350
6	228570	10299	6787	648	2131	228924	207822
7	228843	10436	6868	158	2087	228968	208786
8	228818	10580	6763	144	2802	228253	183593
9	228787	10694	6683	141	2060	228995	211920
15	228679	10580	6910	136	2219	228836	205725
16	228939	10211	7081	74	2181	228874	201762
17	228808	10340	6952	204	2324	228731	200000
19	228973	10057	6998	277	1957	229098	209676

**Figure 3:**
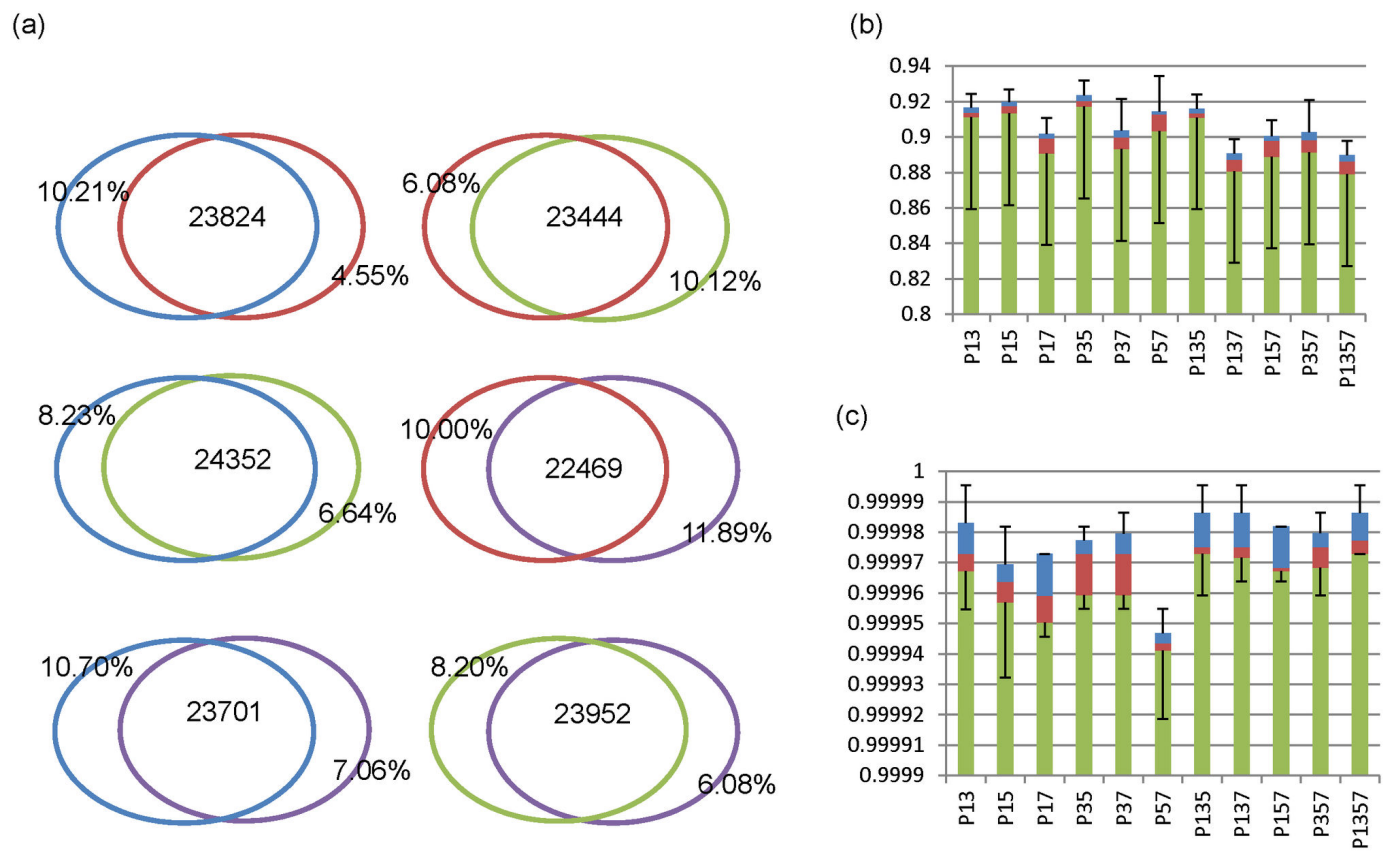
Shared variants by single-sample pipelines and their validation. a. Average pairwise overlapping between filtered variants called by SAMtools (blue), GATK (red), glfSingle (olive green) and Atlas2 (purple). b and c. Boxplots of sensitivities and specificities for shared variants. P13 stands for shared variants between pipeline 1 (SAMtools) and 3 (GATK), P135 stands for shared variants by pipeline 1, 3 and 5 (glfSingle), and so on.

Furthermore, we calculated the pairwise overlapping variants among the four filtered variant sets generated from the single sample-calling pipelines, and obtained six pairs of recapture fractions (fraction of shared variants in each variant set in the pair). Figure 3a shows the average pairwise overlapping for filtered variants. We presented the number of shared variants in the overlapping regions. The most important observation is that the recapture fractions were very high, ranging from 88.11-95.45%, which suggested that the pipelines are in agreement and that our pipelines are reliable. SAMtools had the highest recapture fractions, and GATK had the lowest recapture fractions among the four single-sample calling pipelines. These recapture differences may be attributed to the more stringent featured filtering for GATK and Atlas2, rather than to differences in sensitivity (as shown by validation results above). The shared variants were further compared and validated using the exome array data again. Both the number of TPs and the number of FPs dropped when additional pipelines were used to select shared variants, generating higher specificity and lower sensitivity generally, as shown in Figure 3b and 3c. The highest specificity, 0.9999804±7.79e-6, was attained by taking shared SNPs by all four pipelines (average only 7.5 FPs), while the shared SNPs called by both GATK (single-sample) and glfSingle had the highest sensitivity among these variant sets.

### Sanger Sequencing Results

All 13 variants (six targeted intentionally because they were known to be discrepant, and seven additional variants contained within sequenced amplicons) were detected by Sanger sequencing (see Table S3 in File S1 and the Sanger sequencing results section in the supplementary materials). For the six highly discordant variants, Sanger sequencing confirmed most genotypes called by exome sequencing, with an average genotype (allele) concordance rate of 88.9% (93.8%), compared to 26.4% (40.3%) between the exome array and the Sanger sequencing. For four nearby variants that could be called by Sanger sequencing but that were not included in the exome genotyping array, the average genotype (allele) concordance rate with exome sequencing was 88.2% (94.1%). Genotypes of the other three nearby variants, which had previously been called both from exome arrays and from exome sequencing, were completely concordant with Sanger sequencing results as well.

### Simulated whole genome sequence data

We labeled the five individuals with “mutations” generated by dwgsim-0.1.10 directly from HG19 Chromosome 22 as “Mut01-Mut05.” Table 3 summarizes their “mutant” bases and variants. Averaged over the five, 35,542±207.35 mutant bases were generated, among which the strand one only, strand two only, and both strand mutant bases were distributed evenly (1/3, 1/3, 1/3). These mutant bases defined 34,783±169.2 variants per individual: 31,298±167.4 SNPs and 3,485±70.1 indels.

**Table 3:** Summaries of simulated mutations and variants.

**Subject**	**bases_var**	**strand1**	**strand2**	**both**	**variants**	**SNPs**	**indels**	**%_indels**
Mut01	35650	11918	11923	11809	34829	31247	3582	10.28%
Mut02	35711	11955	11816	11940	34908	31425	3483	9.98%
Mut03	35220	11739	11786	11695	34516	31032	3484	10.09%
Mut04	35447	11758	11845	11844	34728	31344	3384	9.74%
Mut05	35680	11921	11966	11793	34933	31441	3492	10.00%

**Figure 4:**
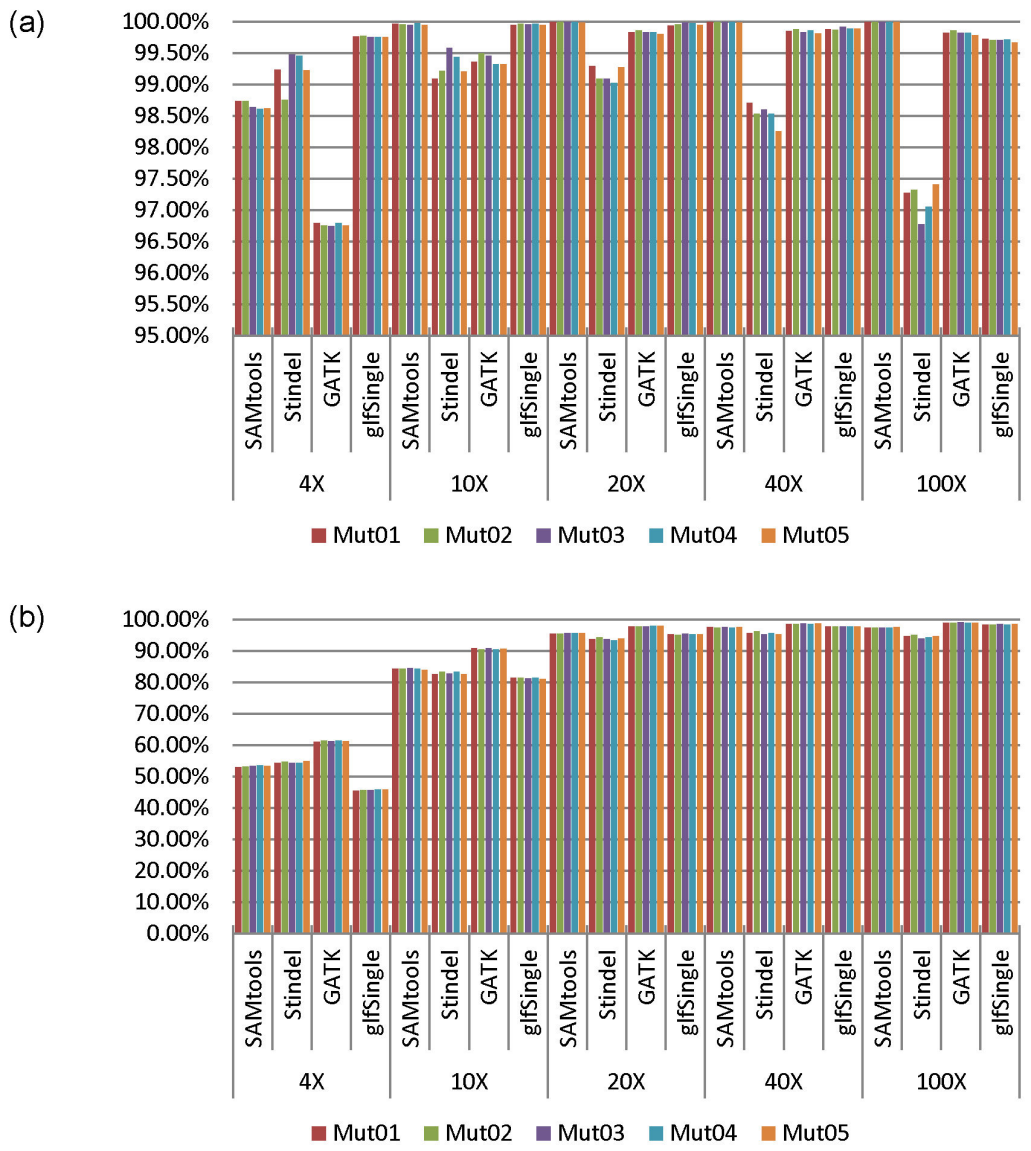
Positive prediction value and sensitivity of callers for WGS data simulated at different coverage settings. The SAMtools, GATK, glfSingle labels represent the sensitivities for SNPs, Stindel represents the sensitivity for indels called by SAMtools. a. Positive prediction value. b. Sensitivity.

We applied the three modified pipelines to the simulated reads (see Table S4 in File S1 for the alignment and coverage summaries). We observed that GATK UnifiedGenotyper failed to call any of these simulated indels. Therefore the variants generated were reduced to four sets: (i) SNPs called by SAMtools, (ii) indels called by SAMtools, (iii) SNPs called by GATK, and (iv) SNPs called by glfSingle. These called variants were compared to the simulated variants using VCFtools-0.1.7 after necessary reformatting, which gave the TPs and FPs directly. The true negatives (TNs) distribute across almost the whole chromosome, and the number of TNs is more than 10,000 times greater than the observed number of false positives (FPs) for all (average) coverage; hence the specificity of each of the three callers was >99.99%. Figure 4 shows the PPV and sensitivity for the four sets (see the simulated whole genome sequence results data section in the supplementary materials (File S1) for more details). At all coverage depths, GATK had a higher sensitivity than SAMtools and glfSingle. SAMtools had higher sensitivity for SNPs than glfSingle at low coverage (4x and 10x), similar sensitivity to glfSingle at 20x, and lower sensitivity than glfSingle at high coverage (40x and 100x). Its sensitivity for indels was close, overall, to its sensitivity for SNPs, but the difference decreased as the coverage depth increased, monotonically, from 1.2% at 4x to -2.93% at 100x. As expected for SNPs, SAMtools had an almost perfect PPV at 20x or higher, and a very high PPV at 10x. However, at 4x, the PPV was only 98.67%, which was lower than glfSingle. GATK gave a lower PPV for SNPs than SAMtools and glfSingle at 4x, 10x, 20x, and 40x, but a higher PPV than glfSingle at 100x. SAMtools’ PPV for indels dropped after 10x. Note that glftools used a hybrid version of SAMtools to calculate first the genotype likelihoods. These results suggest that the majority of the FPs called by GATK at low coverage may be attributed to the algorithm inconsistency between GATK and SAMtools, while the FPs called by SAMtools and glfSingle may mainly be attributed to alignment errors. The influence of that algorithm inconsistency became weaker for SNP detection from high coverage data.

## Discussion

Next-generation sequencing is a powerful tool for identifying rare and *de novo* variants, disease mapping, and quantifying expression levels. For analysis, NGS reads are first aligned to a reference genome, and then subjected to variant calling after necessary quality control procedures. The alignment is crucial for variant calling accuracy, and BWA is a widely-used aligner with good performance. This study evaluated the performance of several popular variant callers, SAMtools, GATK, glftools, and Atlas2, with pipelines incorporating BWA and single-sample or multiple-sample calling strategies. The pipelines were applied to both real whole exome sequencing data and simulated whole genome sequencing data after simplification. The called variants were compared to the exome genotyping array data and simulated variants respectively, both used as “gold standards”. The statistical measures obtained from the exome sequencing data confirmed a widely-accepted idea that filtering is a crucial step for improving the accuracy of variant calling. The exome array data comparison showed that GATK had the highest re-discovery rate (Positive Prediction Value) and specificity, while glftools showed the highest sensitivity. The multiple-sample strategy worked effectively for GATK and glfMultiples, but not for SAMtools. The simulated whole genome sequencing data comparison demonstrated that GATK had higher sensitivity.

GATK performed well on both the real exome sequencing data and the simulated whole genome sequencing data. On the exome sequencing data, it generated the fewest false positives after filtering, and therefore had the highest PPV and specificity. The filtered variants called by GATK had the highest recapture rate compared with SAMtools and glftools. GATK worked with the multiple-sample-calling strategy effectively and avoided inflating false positive genotype calls by assigning missing genotypes. It identified 9.0% more SNPs correctly and doubled the indels calls when using the multiple-sample strategy. For the simulated whole genome sequencing data, GATK had higher sensitivity than SAMtools or glfSingle. The advantage over the latter two was remarkable for low coverage cases. Although there were more false positives with GATK than with SAMtools or glfSingle at very low coverage, the false discovery rate dropped quickly as coverage depth increased. The variants were not filtered. The performance of VQSR and filters for indels on our exome sequence data showed that GATK may also perform well on real whole genome sequencing data after filtering. Based on the results presented herein, it is our recommendation that GATK be used for general purpose NGS analyses, at least for circumstances similar to those we have described.

SAMtools remains a useful tool for many tasks. Because it limits the total read depth to 8000, however, this makes it more suitable to evaluate whole genome sequencing data at moderate coverage rather than target candidate gene sequencing or exome sequencing data, both of which generally contain large portion of sites with higher coverage. On our exome sequencing data, SAMtools missed many variants with multiple-sample calling. Its single-sample detection rate for SNPs was similar to GATK, but lower than glfSingle, while its single-sample detection rate for indels was not systematically different from GATK or Atlas2. When calling indels, SAMtools tended to keep the same tailing bases for both reference and alternate alleles. For example, SAMtools gives TAAAA:TAAA (REFERENCE:ALTERNATE) at Chr1:109486240 for several individuals in our subjects, while GATK and Atlas2 give TA:T which is identified as rs3215062 in dbSNP. This led to more “Non-Matching Reference” cases in comparison with indels called by GATK or Atlas2, and contributed to its lowest average recapture rate among the callers we compared in this study. It called more false positives than GATK. On simulated whole genome sequencing data, SAMtools was not robust to alignment errors at low coverage, and its false discovery rate for indels increased as coverage increased.

Glftools uses a revised version of the SAMtools-0.1.7a-hybrid, which generates genotype likelihood files (GLFs). Glftools calls SNPs from the GLF files rather than the BAM files. A moderate read depth is required for accurate genotype likelihood calculation; so on simulated whole genome sequencing data, glfSingle had low sensitivity at low coverage (4x). On our exome sequencing data, glfSingle and glfMultiples had the highest sensitivity of the approaches we evaluated. The false discovery rate of glfSingle was higher than GATK, but it also called the most TPs. GlfMultiples called significantly more variant sites than GATK and correctly identified a larger number of SNPs (compared to glfSingle) even after filtering. However, the exome array data show that false positives were also rising, mainly due to the non-missing genotype assignment (which was not accurate for cases of low coverage or of close likelihoods). This high sensitivity is an advantage for studies aiming for a high detection rate, and makes glftools a good tool for first round variant discovery.

Atlas2 does not use a traditional probabilistic model method to calculate regular likelihoods; it employs logistic regression models trained to validate whole-exome capture sequencing data. It calls SNPs and indels using separate programs. The Atlas-SNP2 logistic model uses unique variables not included by other callers, such as the mean neighboring base quality (NBQ) around the SNP, the mean distance to the 3’ end, and interaction terms. The Atlas-Indel2 logistic model uses four variables: the local sequence entropy, the strand direction, the normalized variant square, and the mean NBQ. The SNPs and indels can be further filtered heuristically on the posterior probability and read depth, etc. The output of Atlas-SNP2 is still not user-friendly, containing too many raw putative sites and too many whole sequences. Immediate filtering is necessary to make comparable outputs to the other variant callers. Altas-Indel2 allows for interval processing. On our exome sequencing data, Atlas-SNP2 called fewer SNPs (after filtering) than SAMtools and glfSingle, but slightly more SNPs than GATK. Altas-Indel2 called a comparable number of raw indels to SAMtools and GATK, and after filtering, fewer indels than SAMtools but more than GATK. Atlas2 was demonstrably not suitable for analysis of our simulated WGS data (see File S1, last paragraph), because of the estimate failures of specific model parameters which were not taken into simulation. Our conclusion is that filtering on the posterior probability is more stringent than filtering on the variant quality score. However, it is not as stringent as VQSR or the recommended filter set for exome indels. Atlas-Indels generates reference and alternate alleles in a manner consistent with GATK. Therefore, the indels, identified by these two callers, have no allele-matching errors. At the exome array sites, Altas2 calls the fewest TPs among the four and more FPs than GATK. This new caller has been implemented into Genboree Workbench [25] for web-based analysis and has the flexibility to run on a desktop [15]; our data suggest that it has no consistent advantages over the others.

Regarding the computational burden, our pipelines used the same pre-calling procedure such that CPU times for the callers vary for individuals and sampling-strategy for calling. GATK contains multi-thread option itself, which reduced the running time by the number of threads; SAMtools and Atlas2 had longer running time for calling, as they do not contain multi-thread components by themselves; glftools ran slower than SAMtools, as it required the output of the hybrid version of SAMtools. Multiple-sample calling takes much longer than single-sample calling.

We used variant data from the Illumina HumanExome v1.1 Beadchip for validation. High consistency was observed among the variant sets generated by the single-sample pipelines and the real exome sequencing data. For the GATK multiple-sample pipeline, the overall genotype consistency rate (including the 0/0 genotypes) between exome sequencing and the exome array was 99.82%. Although Sanger sequencing in theory is a better method based on real data to generate a “gold standard” of variants for validation, it would not be practical to Sanger-sequence the entire exome. Alternatively, we selected a few variants with high discordance between exome sequencing and the exome array for Sanger sequencing. The results for this highly selected set of variants showed exome sequencing was more accurate for variant genotyping than the exome array, and the high discordance was mainly due to inaccuracy of the exome array rather than exome sequencing. On the other hand, the simulated whole genome data serves as an overall better validation, although it does not correspond to any individual’s actual sequence, because the actual simulated sequence is known without any ambiguity.

In summary, GATK makes a powerful tool for NGS analyses and works effectively with both single-sample and multiple-sample calling strategies. Our results show that it has the highest specificity and PPV on the exome sequencing data and the highest sensitivity on the simulated whole genome sequencing data. Glftools have a higher sensitivity when applied to the exome sequencing data, but produces more false positives than GATK. SAMtools is not especially distinguished for any task except for the high PPV for SNPs on simulated whole genome sequencing data. Atlas2 provides a different method of modeling for variants, but in its current implementation, shows results no better than GATK. Overall, filtering is crucial for improving the accuracy of variants, especially accuracy of indels for which larger variations across pipelines were observed than for SNPs (see Figures S1 and S2). A multiple-sample strategy increases the sensitivity of GATK and glftools, but also increases the number of false positives. For target candidate gene sequencing or exome sequencing studies, our analyses favor the use of GATK. If extremely high specificity is desired, these callers may be used together to define a common intersection of variant sets, especially, glfSingle and GATK together may make a good choice for SNP-calling. For whole genome sequencing at moderate coverage, GATK should be used for first round detection, and SAMtools should be used for recapture to improve confidence.

## Supporting Information

Figure S1 **Raw variants from single-sample callings.** a. Number of raw SNPs. b. Number of raw indels. c. Ti/Tv ratio in raw SNPs.
(TIFF)

Figure S2 **Filtered variants from single-sample callings.** a. Number of filtered SNPs. b. Number of filtered indels. c. Ti/Tv ratio in filtered SNPs.
(TIFF)

Figure S3 **Variants from multiple-sample callings.** a. Number of raw SNPs. b. Number of raw indels. c. Number of filtered SNPs. d. Number of filtered indels.
(TIF)

Figure S4 **Validation of single-sample calling variants using exome array data a.** Number of true positive genotypes. b. Number of false positive genotypes. c. PPV, i.e., rediscovery rate. d. Sensitivity. e. Specificity.
(TIFF)

Figure S5 **Validation of multiple-sample calling variants using exome array data a.** Number of true positive genotypes. b. Number of false positive genotypes. c. PPV, i.e., rediscovery rate. d. Sensitivity. e. Specificity.
(TIF)

File S1 Table **S1**, Summary of validation for variants called from sequencing data in comparison with the exome array data. Table **S2**, Summary of alignment and coverage of exome sequencing data by individual. Table **S3**, Sanger validation for the targeted regions containing discordant variants between exome sequencing and the exome array. Table **S4**, Summary of reads, alignment and coverage for the simulated WGS data.
(DOCX)
