# Supplementary figures and images for: Variant Callers for Next-Generation Sequencing Data: A Comparison Study
Source: PLoS One. 2013 Sep 27;8(9):e75619. doi: 10.1371/journal.pone.0075619 (PMC3785481; doi:10.1371/journal.pone.0075619)

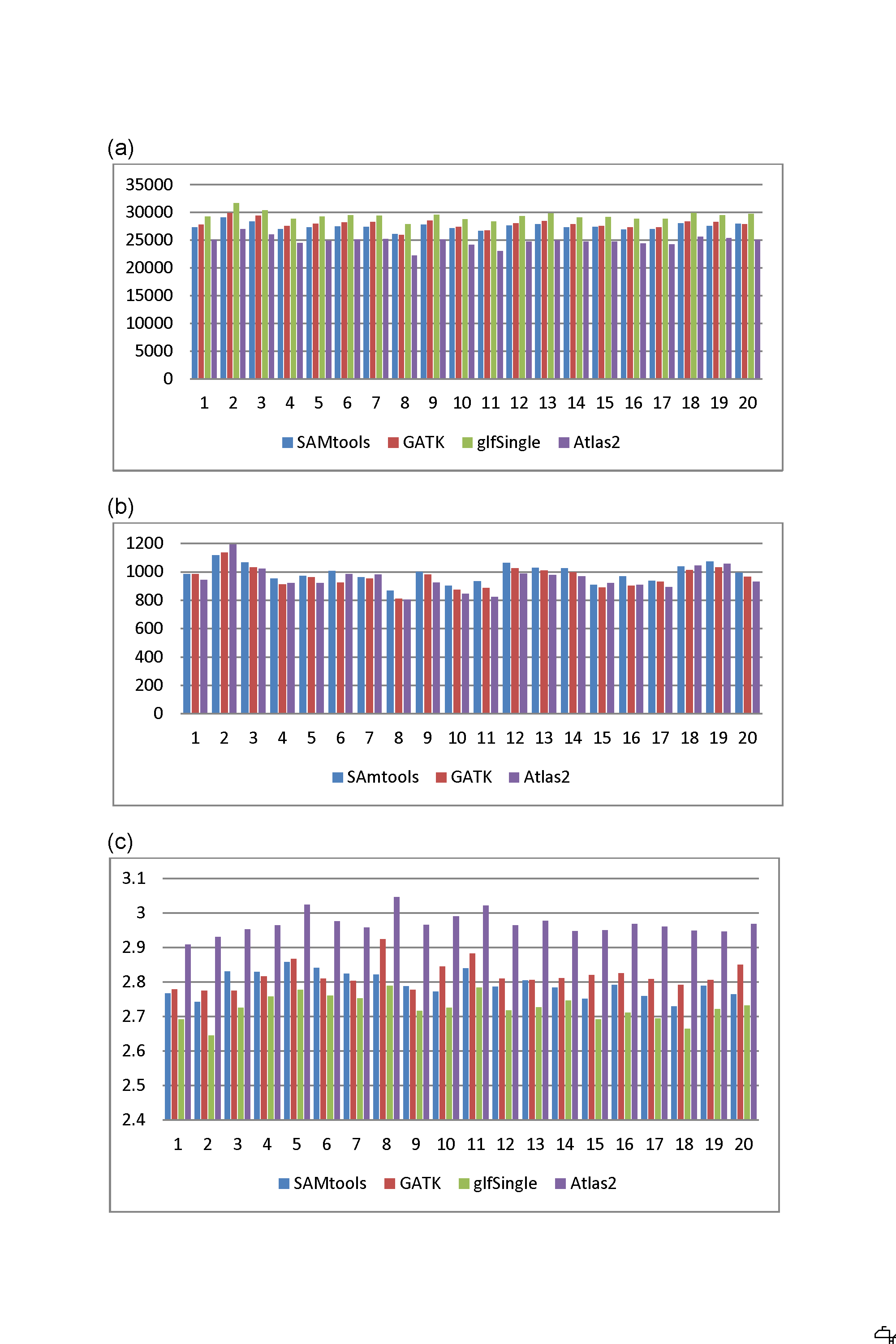

Supplement: Figure S1 — Raw variants from single-sample callings. a. Number of raw SNPs. b. Number of raw indels. c. Ti/Tv ratio in raw SNPs. (TIFF) [file pone.0075619.s001.tiff]

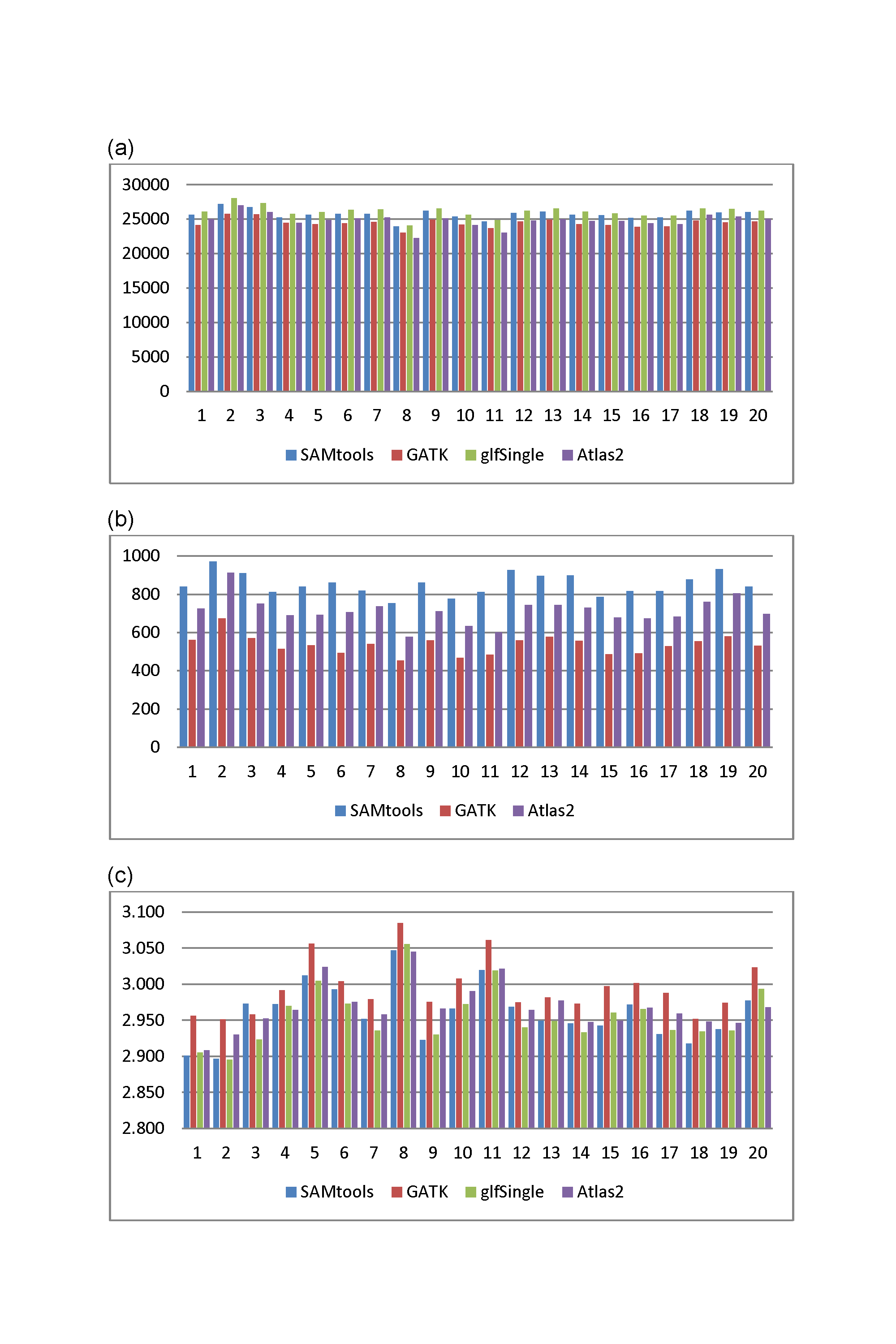

Supplement: Figure S2 — Filtered variants from single-sample callings. a. Number of filtered SNPs. b. Number of filtered indels. c. Ti/Tv ratio in filtered SNPs. (TIFF) [file pone.0075619.s002.tiff]

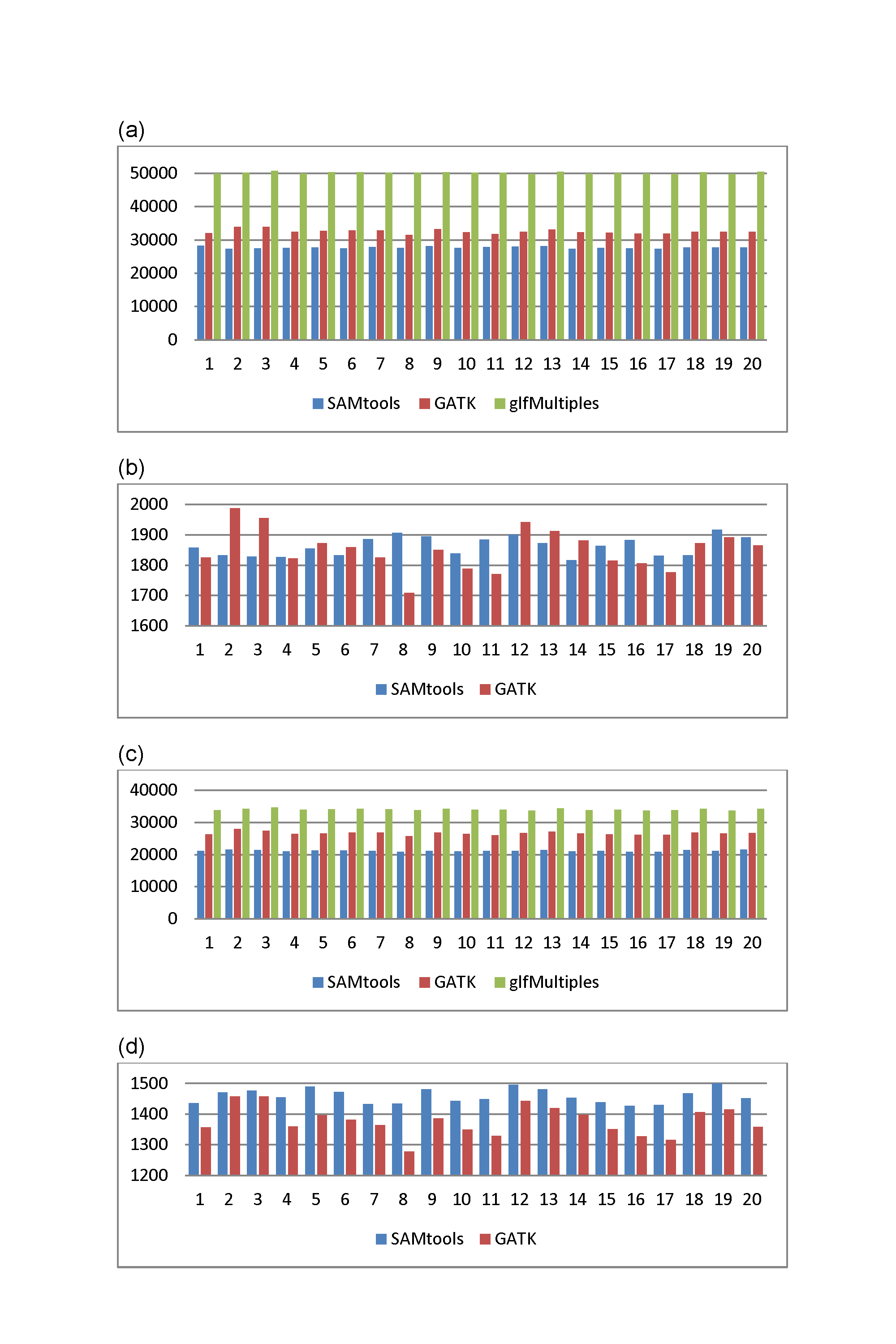

Supplement: Figure S3 — Variants from multiple-sample callings. a. Number of raw SNPs. b. Number of raw indels. c. Number of filtered SNPs. d. Number of filtered indels. (TIF) [file pone.0075619.s003.tif]

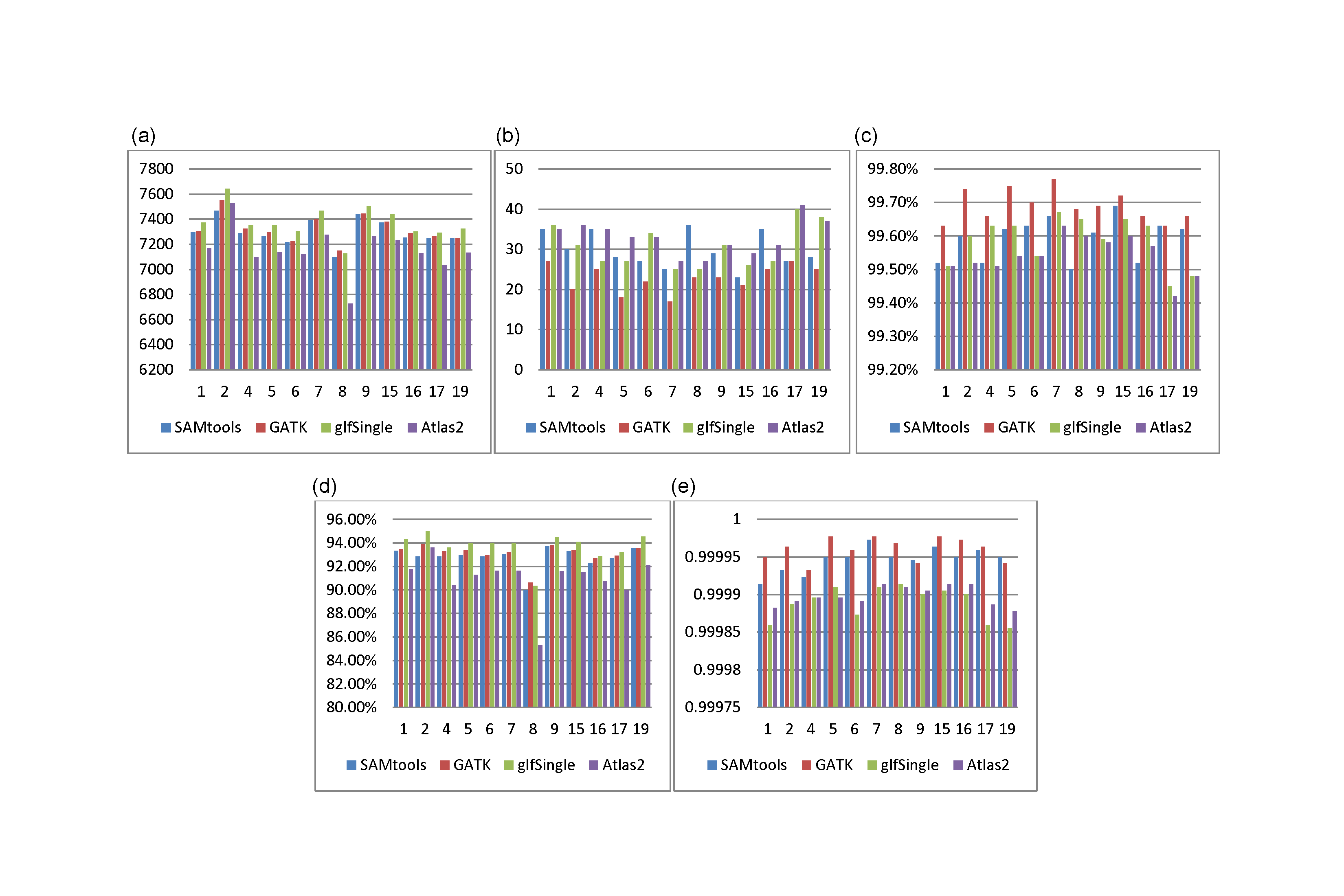

Supplement: Figure S4 — Validation of single-sample calling variants using exome array data a. Number of true positive genotypes. b. Number of false positive genotypes. c. PPV, i.e., rediscovery rate. d. Sensitivity. e. Specificity. (TIFF) [file pone.0075619.s004.tiff]

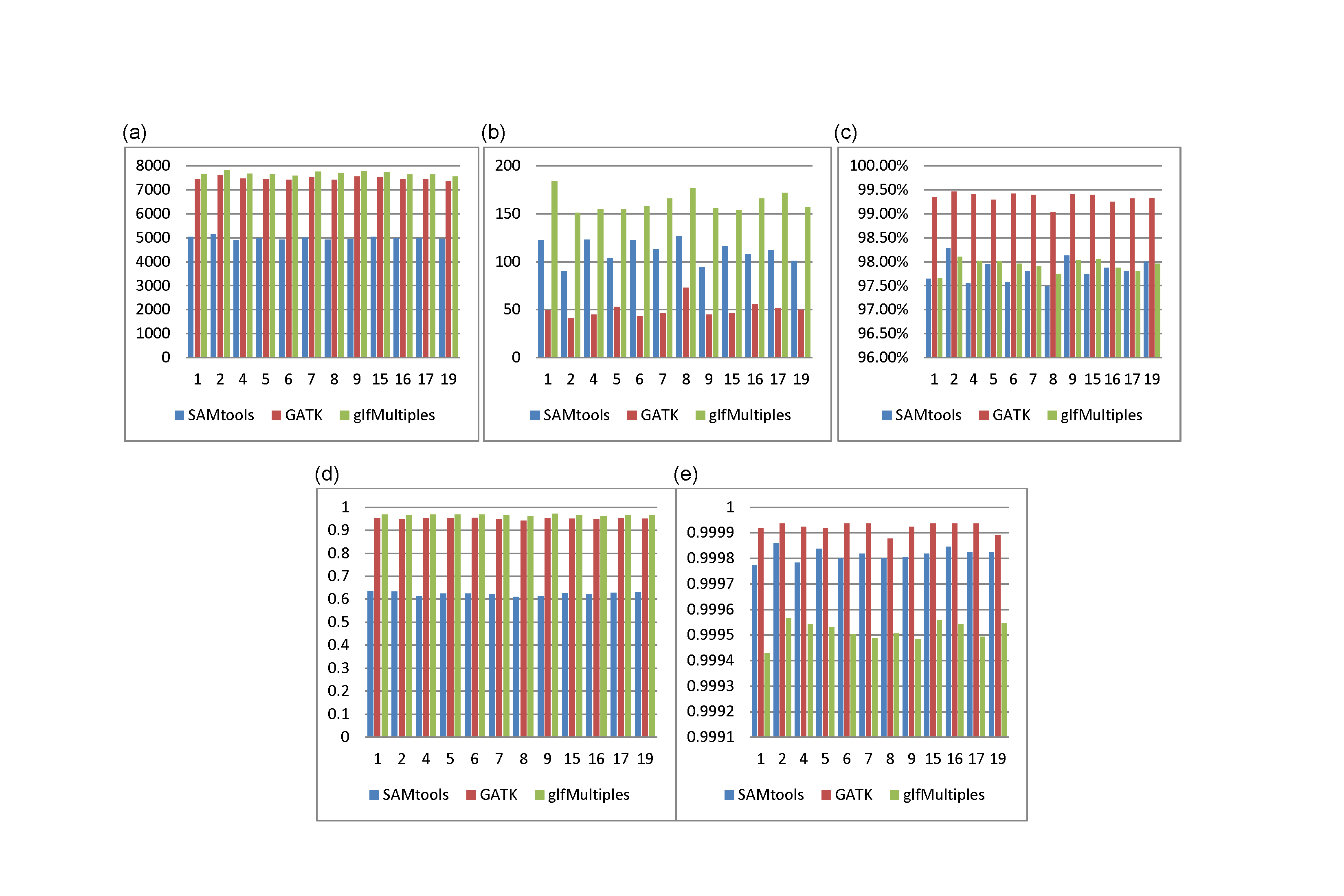

Supplement: Figure S5 — Validation of multiple-sample calling variants using exome array data a. Number of true positive genotypes. b. Number of false positive genotypes. c. PPV, i.e., rediscovery rate. d. Sensitivity. e. Specificity. (TIF) [file pone.0075619.s005.tif]
