# Supplementary material for: Variant Callers for Next-Generation Sequencing Data: A Comparison Study
Source: PLoS One. 2013 Sep 27;8(9):e75619. doi: 10.1371/journal.pone.0075619 (PMC3785481; doi:10.1371/journal.pone.0075619)
Supplement: File S1 — Table S1, Summary of validation for variants called from sequencing data in comparison with the exome array data. Table S2, Summary of alignment and coverage of exome sequencing data by individual. Table S3, Sanger validation for the targeted regions containing discordant variants between exome sequencing and the exome array. Table S4, Summary of reads, alignment and coverage for the simulated WGS data. (DOCX) [file pone.0075619.s006.docx]

**Supplementary Materials**

**Pipeline Implementation**

***Unified pre-calling procedure***

The ‘Unified’ pre-calling procedure is shown in Figure 1 in main text. BWA-0.6.1[1], SAMtools-0.1.18 [2], GATK-1.6-9 [3,4], and Picard-tools-1.53 [5] were implemented. The reads were first mapped to GRCh37/HG19 reference genome downloaded from UCSC by BWA-0.6.1with option ‘-n 2’. Then SAMtools-0.1.18 was used to generate sorted BAM file, which was followed by a mapping quality control (or improvement) process: 1) local realignment by GATK-1.6-9 around known indels in dbsnp132 and 1000 Genomes databases; 2) removal of PCR duplicates by Picard-tools-1.53; 3) base quality score recalibration by GATK-1.6-9 using the dbsnp132 database for training.

***Variant calling***

The BAM files after QC were directly passed to SAMtools-0.1.18, GATK-1.6-9 and Atlas2 v1.0 [6] as input. And Samtools-0.1.7a-hybrid was used to generate genotype likelihood files (GLF) required by glftools [7] as for direct input. The raw variants are called via the following command option settings for the seven pipelines respectively:

1. SAMtools-0.1.18: ‘mpileup -C50’ + ‘vcfutils.pl varFilter -D 10000’
2. SAMtools-0.1.18: ‘mpileup -C50’ + ‘vcfutils.pl varFilter -D 80000’
3. GATK-1.6-9 UnifiedGenotyper ‘-glm BOTH -stand_call_conf 50.0 -stand_emit_conf 10.0 -dcov 200’
4. GATK-1.6-9 UnifiedGenotyper ‘-glm BOTH -stand_call_conf 50.0 -stand_emit_conf 10.0 -dcov 1000’
5. glfSingle: ‘samtools-0.1.7a-hybrid’ + glfSingle default
6. glfMultiples: ‘--minMapQuality 20 --minTotalDepth 60 --maxTotalDepth 10000’
7. Atlas2 v1.0: default

The BED file of exome target regions with 10 bps extension on both ends downloaded from UCSC was used in both GATK (‘-L’) and Atlas-Indel2 (‘-B’) callings.

***Filtering and annotation***

We conducted regional filtering on VCF v4+ files using VCFtools-0.1.7 [8] with the exome BED file, and implemented our own Perl program to complete the task on the VCF v3.3 files generated by glfSingle and glfMultiples.

Quality-based filtering is more complicated. Variant quality threshold was set 20 for variants called by SAMtools-single and glfSingle, and 60 for variants called by SAMtools-multiple and glfMultiples. SNPs by GATK were filtered by the variant quality score recalibration (VQSR) tool VariantRecalibrator using HapMap 3.3, Omni 2.5M chip, and dbsnp132 as training data sets with filter level 99.0. The recommended best practice GATK VariantFiltration filter set (‘QD < 2.0’, ‘ReadPosRankSum < -20.0’, ‘FS > 200.0’) was applied to indels by GATK. For SNPs by Atlas-SNP2, the authors provided a ‘genotyper’ program, and recommended thresholds 0.9 for posterior probability and 8 for read depth. The posterior probability threshold 0.9 was also applied to indels called by Atlas-Indel2. These filtered variant sets were annotated using ANNOVAR [9].

**Exome Array Data Processing**

***Quality control***

The exome array genotype data generated from HumanExome v1.1 Beadchip [10] were given in the format of two forward-strand alleles without the REF/ALT order. The dbsnp132 database was used to fill in allele order and full alternate allele information for multi-allelic sites. We implemented a set of Perl programs to remove duplicate sites, correct strand errors, and conduct the Hardy-Weinberg equilibrium tests. The genotype data after such QC steps were then used to validate the variants called by the pipelines.

***Validation metrics***

Table S1 summarizes the different validation cases for a given individual with bi-allelic sites. For each individual, the variants from the array data can be divided into ten groups: (1) sequencing variants with matched array genotypes; (2) false positive sequencing variants; (3) sequencing variants with non-matched array genotypes; (4) sequencing variants with missing array genotypes; (5) homozygous reference array genotypes without sequencing calls but at least 1x coverage; (6) heterozygous or homozygous alternate array genotypes without sequencing calls but at least 1x coverage; (7) sites with no coverage or outside of the target regions; (8) matched homozygous reference genotypes between sequencing and the array data; (9) homozygous reference genotypes from sequencing data but variant genotypes from the array data; (10) missing genotypes from sequencing data at the matched sites.

Table S1. Summary of validation for variants called from sequencing data in comparison with the exome array data.

| Validation cases | | Genotype from array | | | | |
| --- | --- | --- | --- | --- | --- | --- |
|  |  | None | - | 00 | 01 | 11 |
| Genotype from  sequencing data | None | - | - | TN, (5) & (7) | FN, (6) & (7) | FN, (6) & (7) |
|  | ./. | - | - | (10) | (10) | (10) |
|  | 00 | - | - | TN, (8) | FN, (9) | FN, (9) |
|  | 01 | - | (4) | FP, (2) | TP, (1) | GE, (3) |
|  | 11 | - | (4) | FP, (2) | GE, (3) | TP, (1) |

Variants at chromosomal sites in group (1) are all true positive (TP) predictions, while those at sites in group (2) include the false positive (FP) predictions as well as some true positive variants with genotype errors (GEs) other than ‘0/0’. (5)+(8) and (6)+(9) are the theoretically maximal sets of sites of identifiable true negative (TN) and false negative (FN) variants. Note that variant-calling is trinary in nature. To evaluate the accuracy of variant calling as in binary classification, we did not distinguish the FP and TP variants with GEs in group (2), but rather treat them as false genotype callings. Therefore the rediscovery rate, defined as the proportion of called variants confirmed by exome array genotype data at the matched sites, was given by the positive prediction value (PPV) defined as (1)/((1)+(2)+(3)). The sensitivity and specificity were given by (1)/((1)+(3)+(6)+(9)) and ((5)+(8))/((2)+(5)+(8)) respectively. For single-sample calling pipelines, there were no ‘0/0’ or ‘./.’ in variant outputs, so the sensitivity and specificity were reduced to (1)/((1)+(3)+(6)) and (5)/((2)+(5)). Since the group (6) was larger than the set of practically identifiable FNs, the computed sensitivity was lower than its value, since 1x is far from enough for variant-calling, even not enough for genotyping. Using a higher coverage cutoff point for the identifiable TN and FN sites, we expected the sensitivity would be slightly improved.

**Simulation and Analysis**

***Simulation of WGS data***

The whole genome sequence data were generated using dwgsim-0.1.10 [11], which is upgraded from wgsim [12] initially developed as a part of SAMtools. For computational simplicity, we chose chromosome 22 of reference genome HG19 rather than the whole genome (22 autosomes, 2 sex chromosomes, plus mitochondrion). Five independent mutation sets (individuals) were simulated and saved. 70bp paired-end reads were generated from these mutation sets at average coverage 4x, 10x, 20x, 40x, and 100x. The per base sequencing error for simulating reads was set as 0.02, which is larger than typical empirical average per base sequencing error (~1.3%). By default, rate of mutation was set as 0.001, and fraction of indels among the mutations was set as 0.1.

***Analysis of simulated WGS data***

The ‘Reduced’ pre-calling procedure for the pipelines was applied to the simulated sequencing data. That is, the simulated reads were mapped to reference genome HG19 (chromosome 22 only) by BWA, but the mapping QC process was omitted. Since these mutations were simulated, only three single-sample callings were applied, without filtering or annotation. Note that the mutant variants for each ‘individual’ provide the perfect standard. The PPV and sensitivity (the fraction of simulated variants which were called from the sequence data) surely depend on the coverage and mapping quality threshold. The specificity was not reported, as this measure was nearly one for all three callers, and the differences were tiny. Furthermore, the false positives were not as important as for real sequencing data, since many of which may come from the inconsistency between the algorithms used in the callers and alignment errors. SAMtools is expected to have high PPV, since it is free of algorithm inconsistency for sequencing reads generated by dwgsim as a consequence of its wgsim component. Our focus was on sensitivity.

**Exome Sequencing Results**

The main text shows the summaries of statistical measures. This section presents the corresponding results by individual.

***Unified pre-calling procedure***

The statistics and metrics evaluating the ‘Unified’ part of the pipelines are presented in Table S2.

Table S2. Summary of alignment and coverage of exome sequencing data by individual.

| **Subject** | **read pairs** | **%_mapped** | **%_unique** | **covered_bases** | **mean_coverage** |
| --- | --- | --- | --- | --- | --- |
| 1 | 45121206 | 97.17% | 93.04% | 44051930 | 89.99 |
| 2 | 77635455 | 97.15% | 91.43% | 47808536 | 137.44 |
| 3 | 63493769 | 94.34% | 88.10% | 44956412 | 114.58 |
| 4 | 48400344 | 95.60% | 89.76% | 43615727 | 94.69 |
| 5 | 49562964 | 95.00% | 89.16% | 44175518 | 95.08 |
| 6 | 49739158 | 95.13% | 89.78% | 44150415 | 96.18 |
| 7 | 52253856 | 95.69% | 90.42% | 44380000 | 100.13 |
| 8 | 31867528 | 94.33% | 89.22% | 42642753 | 62.14 |
| 9 | 57724026 | 94.25% | 87.38% | 44445848 | 106.27 |
| 10 | 44786116 | 94.27% | 89.11% | 43592266 | 86.11 |
| 11 | 31006445 | 96.45% | 92.78% | 43047068 | 62 |
| 12 | 52579906 | 96.42% | 91.00% | 44741827 | 98.61 |
| 13 | 53097962 | 96.12% | 90.31% | 44539086 | 99.91 |
| 14 | 50624174 | 96.12% | 91.19% | 44189607 | 97.34 |
| 15 | 43161151 | 96.19% | 91.61% | 43674918 | 87.21 |
| 16 | 39086817 | 96.43% | 92.10% | 43953151 | 77.58 |
| 17 | 39440982 | 97.07% | 92.83% | 43746698 | 78.84 |
| 18 | 52546059 | 96.53% | 90.01% | 44299710 | 102.97 |
| 19 | 49532027 | 97.06% | 92.61% | 44726413 | 96.17 |
| 20 | 42491308 | 97.04% | 92.70% | 44178217 | 84.08 |

***Single-sample calling***

Figure S1 shows the numbers of raw SNPs, indels and Ti/Tv ratio for the raw SNPs on target for each single sample calling pipeline across 20 subjects. An exception was the SNPs called by Atlas-SNP2 that was filtered already. On average, SAMtools called 27.45±0.64k SNPs and 990±64 indels, with Ti/Tv ratio 2.79; GATK called 27.92±0.85k SNPs and 961±73 indels, with Ti/Tv ratio 2.82; GlfSingle called 29.35±0.79k SNPs, with Ti/Tv ratio 2.73; Atlas2 called 24.80±0.98k SNPs and 953±87 raw indels, with Ti/Tv ratio 2.97. Overall, SAMtools called about 1.7% less raw SNPs than GATK and generated a close Ti/Tv ratio, while glfSingle called 5.1% more SNPs on target than GATK but the Ti/Tv ratio was much lower. This suggests that glfSingle has a higher detection rate than GATK and SAMtools for SNPs, while the detection rates of the latter two callers are close. No systematic differences were found for numbers of raw indels.

Figure S2 shows the number of filtered SNPs, indels, and Ti/Tv ratio for the filtered SNPs for each single sample calling pipeline across 20 subjects. On average, the filtering process removed 6.4%, 12.5% and 11.1% of the SNPs, and 13.9%, 44.2% and 25.2% of indels for the SAMtools, GATK and glfSingle, respectively. Numbers of SNPs called by the four pipelines were overall close, in the range 24.4-26.1k per sample on average. More specifically, glfSingle called the most SNPs, SAMtools identified more of the filtered SNPs than GATK and Atlas2. This suggests that glfSingle has the highest detection rate for SNPs. There is no systematic pattern for number of filtered indels too. The average Ti/Tv ratios after filtering were 2.96 (SAMtools), 2.99 (GATK), and 2.96 (glfSingle), which are similar and closer to the expected value 3.2 for known SNPs. Indeed, GATK gave slightly higher Ti/Tv ratios for every single individual. Filtering played a key role to improve accuracy of variants, raised Ti/Tv ratio from 2.73-2.82 to 2.96-2.99. This suggests that a larger portion of transversions than transitions be of low quality.

***Multiple-sample calling***

For the 20 individuals together, SAMtools called 70,763 SNPs and 3,619 indels in target regions, GATK called 86,592 SNPs and 3,817 indels, and glfMultiples called 106,775 SNPs. The variants were summarized by individual. Figures S3a and S3b show the numbers of raw SNPs and indels. On average, glfMultiples called 54.16% more raw SNPs than GATK and 81.21% more raw SNPs than SAMtools. There was no systematic pattern between SAMtools and GATK for number of raw indels. Figure S3c and S3d show the numbers of SNPs and indels after filtering. On average, glfMultiples generated 27.83% more filtered SNPs than GATK and 60.86% more than SAMtools. We could conclude that glfMultiples has a significantly higher SNP-detecting rate than GATK and SAMtools. For all individuals, SAMtools generated more filtered indels than GATK. This again shows that the filters applied to GATK indels are more stringent than filtering on variant quality score.

Multiple-sample calling pipelines are expected to call more variants than the corresponding single-sample calling pipelines. SAMtools multiple-sample calling generated almost the same number of raw SNPs as single-sample calling (with an average increment 0.87%), GATK multiple-sample calling generated 16.56% more raw SNPs than single-sample calling, while glfMultiples called 70.96% more raw SNPs than glfSingle. For raw indels, the increment percentages were 88.20% and 92.63% for SAMtools and GATK respectively. After filtering, GATK multiple-sample calling gained 9.02% of SNPs and 157.00% indels over single-sample calling, glfMultiples gained 30.50% SNPs over glfSingle, and SAMtools multiple-sample calling also gained 70.25% indels over single-sample calling. However, SAMtools surprisingly lost 17.60% SNPs on average. This suggests SAMtools multiple-sample calling does not work well for whole exome sequence data. In fact, the SAMtools mpileup command sets max per-file depth to 400 in a mandatory manner, which may account for this poor performance. We can conclude that multiple-sample strategy has much stronger impact on indel detection than on SNP detections, and its power to raise SNP-detecting rate over single-sample calling is strong for GATK, and extremely strong for glftools.

**Exome Array Results**

We had genotypes of 12 individuals for 247,134 markers, which included 829 duplicate sites according to chromosomal positions. That is, we had 246,305 unique sites, 246,167 SNP sites and 138 indel sites. Table 1 in the main text presents genotype summary for the individuals as well as the sequencing coverage on these sites which were extracted from the corresponding ready-to-call BAM files. At the 246,305 unique sites, the 12 individuals had 228,816.17±135.75homozygous reference genotypes, 10,370.92±190.86 heterozygous genotypes, 6,903.08±116.17 homozygous alternate genotypes, and 214.83±148.18 missing genotypes. Especially, no variant genotypes were found at 214,518sites, i.e., for the vast majority (87.09%) of the array markers, the 12 individuals had only homozygous reference or missing genotypes.

The exome array data were used to check the rediscovery rate of the variants generated by the pipelines. This rediscovery rate depends on the coverage at the sites in the array data. The BED file containing exome target regions was used again. Among the 246,305 unique sites, 15,250 were outside of the target regions, and therefore excluded for further analyses. For the 231,055 sites, on average 2,149.5±291.6 sites were not covered by any reads, and 205,630±8,447.9 sites had at least 20x coverage. This suggests the DNA capture was completed similarly well for each individual, but there are remarkable coverage variations across individuals.

For the variants called via single-sample pipelines, Figure S4 shows the number of called variants confirmed by the exome array data, the number of called variants different from array genotypes, and the rediscovery rate. On average, 7,299±104 variants called by SAMtools were validated by the array, while 30±4.4 variants were not. These numbers were 7324±108 and 23±3.3 for GATK, 7373±129 and 31±5.3 for glfSingle, 7154±185 and 33±4.2 for Atlas2. The average rediscovery rate was 99.59%, 99.69%, 99.59% and 99.54% for SAMtools, GATK, glfSingle and Atlas2, respectively. For 11 of the 12 individuals, glfSingle generated the most TPs with one exception, in which glfSingle called 22 true variants less than GATK. GATK generated the least FPs and the highest rediscovery rate. GlfSingle had the highest sensitivity 93.70%, and GATK had the highest specificity 0.99996. Note that glfSingle calls only SNPs. We conclude that GATK has the highest positive prediction value and specificity, and glfSingle has the highest sensitivity.

For the variants called via multiple-sample pipelines, Figure S5 shows the number of true positive predictions, the number of variant predictions different from array genotypes, the number of true negative predictions and the rediscovery rate. GATK called variants on 20,637 array variants, glfMultiples called 20,927 variants, while SAMtools called only 15,635 variants. On average, 7,474±70.9 variants and 12,922±55.6 ‘0/0’ genotypes called by GATK were validated by the data from array, while 50±8.5 variants called were not validated, and 36±14 ‘0/0’ were false negatives. These numbers were 7,681±75.4, 12,982±56.6, 163±10.4 and 49±16.6 respectively for glfMultiples, and 4,980±66.1, 10,426±51.3, 111±11.9 and 82±22.8 respectively for SAMtools. Compared to single-sample calling, GATK multiple-sample calling detected 2.04% more validated variants, and more than doubles the false positive genotype calls. GlfMultiples detected 4.18% more true positives than glfSingle, and raised false positive calls by a factor of 5.32. SAMtools multiple-sample calling missed 1/3 TPs by single-sample calling, even though numbers of FPs were increased. The average rediscovery rate was 97.82%, 99.34%, and 97.93% for SAMtools, GATK, and glfMultiples respectively. The much lower true positive/negative detection rates, and higher false positive/negative rates, show that SAMtools multiple-sample calling fails for many of the variant sites. Within the other two, glfMultiples called 2.77% more true positives and 0.46% more true negatives than GATK, and 226.27% more false positives and 36.11% more false negatives. Again, GATK had higher PPV and specificity, while glfMultiples had the slightly higher sensitivity.

**Sanger Sequencing Results**

We chose 6 exonic variants obtained by the GATK multiple-sample calling pipeline with discordance rate of at least 2/3, namely set (a): SNPs rs12040910, rs1060878, rs654686, rs348942, insertion rs71082910 and novel deletion chr13: 46170719. Then we extended from the selected variants in both 5’ and 3’ directions to target regions of about 400 bps for Sanger sequencing, which in each case covered the exon start position. The PCR products generated for sequencing also included a total of 7 other nearby variants, namely set (b): SNPs rs7413442, rs1140952, rs17412418 and insertion rs35336557 from exome sequencing but not included in the exome array; and set (c): SNPs rs348943, rs3014939, rs17066954 with complete concordance. These 13 variants were evaluated by Sanger sequencing, but the non-variant sites with all 0/0 genotypes in the arrays were excluded.

Table S3. Sanger validation for the targeted regions containing discordant variants between exome sequencing and the exome array.

| **GENE** | | **SEC16B** | **CTAGE5** | **KIR3DL2** | **MCC** | **FAM194B** | **WDR66** | **SEC16B** | **CTAGE5** | **KIR3DL2** | **MCC** | **MCC** | **FAM194B** | **FAM194B** |
| --- | --- | --- | --- | --- | --- | --- | --- | --- | --- | --- | --- | --- | --- | --- |
| **Variant** | | **rs12040910** | **rs1060878** | **rs654686** | **rs348942** | **chr13:**  **46170719** | **rs71082910** | **rs7413442** | **rs1140952** | **rs17412418** | **rs35336557** | **rs348943** | **rs3014939** | **rs17066954** |
| **REF** | | **T** | **G** | **G** | **T** | **C...T** | **G** | **G** | **A** | **C** | **T** | **C** | **C** | **A** |
| **ALT** | | **C** | **A** | **A** | **C** | **C** | **G...A** | **A** | **G** | **G** | **TGCC** | **T** | **T** | **T** |
| **Sample** | **1** | **0/1:TT:TC** | **0/1:AA:AG** | **0/1:GG:GG** | **0/1:TT:TC** | **0/1:CC:D0** | **1/1:GG:II** | **0/0:.:GG** | **0/0:.:AA** | **0/1:.:GG** | **0/1:.:TT** | **0/1:TC:CT** | **0/1:TC:TC** | **0/0:AA:AA** |
|  | **2** | **1/1:TT:CC** | **1/1:AA:AA** | **0/1:GG:AG** | **1/1:TT:CC** | **0/1:CC:D0** | **0/1:GG:GG** | **0/1:.:AG** | **0/0:.:AA** | **0/0:.:CC** | **0/0:.:IT** | **0/0:CC:CC** | **0/1:TC:TC** | **0/0:AA:AA** |
|  | **4** | **0/1:TT:TC** | **0/0:AA:GG** | **1/1:GG:AA** | **1/1:TT:CC** | **0/0:CC:00** | **1/1:GG:II** | **0/0:.:GG** | **0/1:.:AG** | **0/0:.:CC** | **1/1:.:II** | **1/1:TT:TT** | **0/0:CC:CC** | **0/1:TA:AT** |
|  | **5** | **1/1:TT:CC** | **0/0:AA:GG** | **0/0:GG:GG** | **0/1:TT:CT** | **0/1:CC:D0** | **1/1:GG:II** | **0/1:.:AG** | **0/1:.:AG** | **0/0:.:CC** | **0/0:.:TT** | **0/0:CC:CC** | **0/1:TC:TC** | **0/0:AA:AA** |
|  | **6** | **0/1:TT:TT** | **1/1:AA:AA** | **0/1:GG:GG** | **1/1:TT:CC** | **1/1:CC:DD** | **0/1:GG:II** | **0/0:.:GG** | **0/0:.:AA** | **0/0:.:CC** | **1/1:.:II** | **1/1:TT:TT** | **1/1:TT:TT** | **0/0:AA:AA** |
|  | **7** | **1/1:TT:CC** | **0/0:AA:GG** | **0/0:GG:GG** | **0/0:TT:TT** | **0/1:CC:D0** | **1/1:GG:II** | **0/0:.:GG** | **0/1:.:AG** | **0/0:.:CC** | **0/0:.:TT** | **0/0:CC:CC** | **0/1:TC:TC** | **0/1:TA:AT** |
|  | **8** | **0/1:TT:TC** | **0/1:AA:AG** | **0/1:GG:GG** | **./.:TT:CC** | **0/1:CC:D0** | **1/1:GG:II** | **0/0:.:GG** | **0/0:.:AA** | **0/0:.:CC** | **0/0:.:TT** | **1/1:TT:TT** | **0/1:TC:TC** | **0/1:TA:AT** |
|  | **9** | **1/1:TT:CC** | **0/1:AA:AG** | **0/1:GG:GG** | **1/1:TT:CC** | **0/1:CC:D0** | **1/1:GG:II** | **0/0:.:GG** | **0/1:.:AG** | **0/0:.:CC** | **./.:.:TT** | **0/1:TC:CT** | **0/1:TC:TC** | **0/1:TA:AT** |
|  | **15** | **1/1:TT:CC** | **0/1:AA:AG** | **0/0:GG:GG** | **0/0:TT:TT** | **0/1:CC:D0** | **1/1:GG:II** | **1/1:.:AA** | **1/1:.:GG** | **0/1:.:CG** | **./.:.:TT** | **0/0:CC:CC** | **0/1:TC:TC** | **0/0:AA:AA** |
|  | **16** | **1/1:TT:CC** | **0/0:AA:GG** | **0/0:GG:GG** | **0/1:TT:TC** | **0/0:CC:00** | **1/1:GG:II** | **0/0:.:GG** | **0/1:.:AG** | **0/1:.:GC** | **0/0:.:TT** | **0/1:TC:CT** | **0/0:CC:CC** | **0/0:AA:AA** |
|  | **17** | **1/1:TT:CC** | **0/1:AA:AG** | **0/1:GG:GG** | **1/1:TT:CC** | **0/1:CC:D0** | **1/1:GG:II** | **0/0:.:GG** | **0/1:.:AG** | **0/1:.:GG** | **./.:.:II** | **1/1:TT:TT** | **0/1:TC:TC** | **0/0:AA:AA** |
|  | **19** | **1/1:TT:CC** | **0/0:AA:GG** | **0/1:GG:GG** | **1/1:TT:CC** | **0/0:CC:00** | **1/1:GG:II** | **0/1:.:AA** | **0/1:.:AG** | **0/0:.:CC** | **1/1:.:II** | **0/1:TC:TC** | **0/0:CC:CC** | **0/1:TA:AT** |
| **ES vs. EA** | | **0.00%** | **16.67%** | **33.33%** | **18.18%** | **25.00%** | **0.00%** | **.** | **.** | **.** | **.** | **100.00%** | **100.00%** | **100.00%** |
| **ES vs. SS** | | **91.67%** | **100.00%** | **50.00%** | **100.00%** | **100.00%** | **91.67%** | **91.67%** | **100.00%** | **83.33%** | **77.78%** | **100.00%** | **100.00%** | **100.00%** |
| **EA vs. SS** | | **8.33%** | **16.67%** | **83.33%** | **16.67%** | **25.00%** | **8.33%** | **.** | **.** | **.** | **.** | **100.00%** | **100.00%** | **100.00%** |

Note: Genotype (GT) is displayed as: "Exome Sequencing GT: Exome Array GT: Sanger Sequencing GT. ES, Exome Sequencing; EA, Exome Array; SS, Sanger Sequencing. C…T = CCCAGATACTCTTCCTCCT; G…A = GGAGGAGGAGGAGAAA

The Sanger sequencing data were analyzed using CodonCode Aligner [13]. All 13 variants were detected by the Sanger sequencing (Supplementary Table S3). The results are as follows:

1. For the six discordant variants in set (a), the average genotype (allele) concordance rates was 88.9% (93.8%) between exome sequencing and Sanger sequencing, compared to 26.4% (40.3%) between exome array and Sanger sequencing. More specifically, complete concordance between exome sequencing and Sanger sequencing was observed for SNPs rs1060878, rs348942, and the novel deletion (chr13: 46170719). High concordance between exome sequencing and Sanger sequencing was observed for SNP rs12040910 (91.7%) and insertion rs71082910 (91.7%). However, SNP rs654686 was an exception, for which the genotype (allele) concordance rates between exome sequencing and Sanger sequencing was 50% (75%), while the concordance rates between exome array and Sanger sequencing was 83.3% (87.5%). This should not be interpreted as exome sequencing performed worse than the exome array for this SNP. In fact, exome sequencing successfully identified the two variant genotypes, but called 6 heterogyzous genotypes, which correspond to the homozygous genotypes from the reference Sanger sequencing, while exome array failed to identify any variant alleles (Supplementary Table S3). The NCBI SNP database shows that the minor allele frequency is 0.5 for European American, but the sample size is only two.
2. For variants in set (b), the average genotype (allele) concordance rate was 88.2% (94.1%) between exome sequencing and Sanger sequencing. Complete concordance was observed for rs1140952.
3. For SNPs in set (c), exome sequencing and exome array yielded completely concordant genotypes, which were validated by Sanger sequencing at 100% concordance rate.

In summary, the Sanger sequencing confirms most genotypes called by exome sequencing for the highly discordant variants. Exome sequencing is a more accurate method for variant genotyping than the exome array. Exome array is economical, but cannot detect indels/structural variations in most cases, and is less accurate than full exome sequencing. Discordant results (based on our small sample) may be due to inaccuracy in the arrays rather than in the sequencing. By correcting these discordant genotypes, the specificity of our pipelines would be further raised to nearly perfect.

**Simulated Whole Genome Sequence Results**

HG19 Chromosome 22 contains 51,304,566 bases in total, but the first 16,050,000 bases are not determined and marked by ‘N’. With the default settings described above, dwgsim-0.1.10 generated 35-36 k mutation bases each time. Table 2 in main text summarizes the simulated mutant bases and variants. The five simulated mutant individuals were labeled by ‘Mut01-Mut05’. On average over the five individuals, 35542±207.35 mutant bases were generated, among which the strand one only, strand two only, and both strand mutant bases were distributed nearly (1/3, 1/3, 1/3). These mutant bases defined 34783±169.2 variants per individual: 31298±167.4 snps and 3485±70.1 indels. That is, 10.02% of the variants was indels. The simulated base mutation rate was very close to the set parameter 0.001 for all the five individuals, with relative error rates no more than 1.3%. And the indel/variant ratio was within 2.8% and around 0.1.

Table S4. Summary of reads, alignment and coverage for the simulated WGS data.

| **Subject** | **cov_gen** | **Read_pairs** | **%_mapped** | **bases>=1x** | **mean_depth** |
| --- | --- | --- | --- | --- | --- |
| Mut01 | 4x | 1542994 | 89.38% | 34739753 | 5.51807 |
|  | 10x | 3857486 | 89.36% | 34894178 | 13.7328 |
|  | 20x | 7714972 | 89.36% | 34894359 | 27.4604 |
|  | 40x | 15429945 | 89.36% | 34894402 | 54.9281 |
|  | 100x | 38574862 | 89.36% | 34894417 | 137.306 |
| Mut02 | 4x | 1542994 | 89.33% | 34740550 | 5.51455 |
|  | 10x | 3857486 | 89.33% | 34894149 | 13.7264 |
|  | 20x | 7714972 | 89.35% | 34894333 | 27.4598 |
|  | 40x | 15429945 | 89.38% | 34894406 | 54.9348 |
|  | 100x | 38574862 | 89.35% | 34894419 | 137.298 |
| Mut03 | 4x | 1542994 | 89.40% | 34739603 | 5.51894 |
|  | 10x | 3857486 | 89.36% | 34894157 | 13.7313 |
|  | 20x | 7714972 | 89.38% | 34894315 | 27.4703 |
|  | 40x | 15429945 | 89.38% | 34894406 | 54.9396 |
|  | 100x | 38574862 | 89.36% | 34894418 | 137.308 |
| Mut04 | 4x | 1542994 | 89.40% | 34739184 | 5.51914 |
|  | 10x | 3857486 | 89.35% | 34894213 | 13.7304 |
|  | 20x | 7714972 | 89.36% | 34894355 | 27.4612 |
|  | 40x | 15429945 | 89.36% | 34894387 | 54.9271 |
|  | 100x | 38574862 | 89.36% | 34894417 | 137.302 |
| Mut05 | 4x | 1542994 | 89.39% | 34738875 | 5.51828 |
|  | 10x | 3857486 | 89.38% | 34894180 | 13.7345 |
|  | 20x | 7714972 | 89.36% | 34894355 | 27.4632 |
|  | 40x | 15429945 | 89.35% | 34894388 | 54.9204 |
|  | 100x | 38574862 | 89.35% | 34894421 | 137.293 |

Table S4 shows the summary of the reads, alignment and coverage information. The total number of simulated reads was fixed for given average coverage depth: 3,085,988 for 4x, 7,714,972 for 10x, 15,429,944 for 20x, 30,859,890 for 40x, and 77,149,724 for 100x. These suggest that the number of reads be determined according to the whole 51.3 million bases rather than the 35.3 million ATCGs. On average 89.36±0.02% of the reads was aligned to the reference genome. This is lower than the mapped percentage 95.92±1.04% for real exome sequencing data, mainly attributed to the long ‘N’ sequence at the beginning of the reference. Even with the relatively lower mapped percentage, the mean coverage depth was still about 37% larger than the preset values: 5.52 for 4x, 13.73 for 10x, 27.46 for 20x, 54.93 for 40x, and 137.30 for 100x. These mapped percentages and mean coverage depths show that BWA performed with extremely high consistency on these simulated reads.

SAMtools, GATK and glfSingle were used to call the variants. An important observation was that GATK UnifiedGenotyper did not call these simulated indels. Therefore the variant sets generated were: (i) SNPs called by SAMtools, (ii) indels called by SAMtools, (iii) SNPs called by GATK, and (iv) SNPs called by glfSingle. For the 4x simulation, these four sets had size 16,921±128.9, 1,917±42.4, 19,835±139.6, 14,344±113.3 on average. That is, GATK called significantly more SNPs than SAMtools, and glfSingle called the least. For the 10x simulation, the four numbers were 26,394±119.7, 2,912±53.4, 28,553±129.3, and 25,462±136.4. For the 20x simulation, the four numbers were 29,916±156.2, 3,299±69.6, 30,686±169.4, and 29,842±144.5. The order of the numbers did not change. For 40x and 100x simulations, GATK still called the most SNPs, 30,924±160.1 and 31,044±154.6 respectively. However, glfSingle exceeded SAMtools in SNP detection, giving 30,661±167.6 and 30,989±165.9 over 30,519±159.7 and 30,508±161.7 by SAMtools respectively. With higher coverage, GATK and glfSingle called more SNPs. SAMtools showed similar feature at 4x, 10x, 20x, and 40x. However, at 100x, it called more SNPs than 40x for two individuals Mut01 and Mut02, but less SNPs than 40x for Mut03-Mut05.

These called variants were compared to the simulated variants after necessary reformatting. Filtering was not included, as the powerful variant quality score recalibration is not applicable for randomly simulated variants. The reformatting step unified the site position and allele coding for indels, and corrected headlines for SNPs called by glfSingle. After that, the comparisons were easily done using VCFtools-0.1.7, which gave directly the TPs and FPs. Note that the TNs were nearly on the whole chromosome, so the specificity was surely greater than 99.99%. The TPs for the four variant sets (i)-(iv) at 4x were 16,695±122.0, 1,902±39.6, 19,194±134.6, and 14310±112.7 respectively, while the FPs were 225±11.2, 15±5.7, 641±6.7 and 34±1.3. Numbers of TPs in all sets was increased 47.9-77.9% from 4x to 10x, 7.9-17.2% from 10x to 20x, and 0.79-2.67% from 20x to 40x. Between 40x to 100x, number of TPs for (i) stayed almost the same, number of TPs in (ii) even dropped by 1.2%, while numbers of TPs in (iii) and (iv) rose 0.36% and 0.62%, respectively. Number of FPs in (i) dropped dramatically to 10.6±2.6 at 10x, and remained almost zero at 20x or higher, while number of FPs in (ii) kept rising steadily with increasing coverage. Number of FPs in (iii) dropped to 174±22.2 at 10x, and reached a plateau around 50 at 20x or higher. Number of FPs in (iv) dropped to 10±2.3 at 10x, stayed around as 11±5.7 at 20x, but rose after to 34±6.5 at 40x and 90±7.6 at 100x. From the results, we computed the positive prediction value (PPV) and sensitivity, and focused on the latter. Figure 4 shows the PPV and sensitivity for the four sets. GATK showed higher sensitivity at all coverage depth than SAMtools and glfSingle. SAMtools had higher sensitivity for SNPs than glfSingle at low coverage (4x and 10x), similar sensitivity to glfSingle at 20x, and lower sensitivity than glfSingle at high coverage (40x and 100x). Its sensitivity for indels was overall close to its sensitivity for SNPs, but their difference dropped monotonely from 1.2% at 4x to -2.93% at 100x. As expected for SNPs, SAMtools had almost perfect PPV at 20x or higher, and very high PPV at 10x, but 98.67% at 4x, which was lower than glfSingle. GATK gave lower PPV for SNPs than SAMtools and glfSingle at 4x, 10x, 20x, and 40x, but higher PPV than glfSingle at 100x. SAMtools’s PPV for indels dropped after 10x. Note that glftools use a hybrid version of SAMtools to calculate the genotype likelihoods first. These results suggest that the majority of the FPs called by GATK at low coverage may attribute to the inconsistency between the algorithms, while the FPs called by SAMtools and glfSingle may mainly attribute to alignment errors. The influence of algorithm inconsistency became weaker for SNP detection from high coverage data.

In order to replicate these results, we repeat the simulations using chromosome 12, which contains 133,851,895 bases and the preceding ‘N’-bases are less than 60,000. The results were almost the same.

Atlas2 was also used to call variants from the simulated WGS data. Atlas-SNP2 identified no SNPs with posterior probability at least 0.5 at depth 4x, 10x, 20x and 40x, and 2.14±0.04% SNPs at depth 100x; while the average number of raw indels called by Atlas-INDEL2 was increased from 7 (0.2%) at 4x, through 104 (3.0%) at 10x, 434.4 (12.5%) at 20x, 688.6 (19.8%) at 40x, to about 1069.2 (30.7%) as the depth increased. This is not surprising since that Atlas2 employs logistic models with biologically relevant parameters such as mean neighboring base quality (NBQ) around the SNP, the mean distance to the 3’ end, the local sequence entropy, the normalized variant square, etc. Our simulation did not consider such parameters, e.g. NBQ was a constant and the local sequence entropy was close to zero in WGS data. In addition, the parameter estimates used in Atlas2 are trained on validated whole exome sequencing data. Such estimates may not be applicable to real WGS data without further training. Therefore these variants were not compared to the simulated variants for validation.

**Data Release Issue**

Due to human subjects’ issues, we are unable to deposit the data in a repository.

**Reference**

1. Li H, Durbin R (2009) Fast and accurate short read alignment with Burrows-Wheeler transform. Bioinformatics 25: 1754-1760.

2. Li H, Handsaker B, Wysoker A, Fennell T, Ruan J, et al. (2009) The Sequence Alignment/Map format and SAMtools. Bioinformatics 25: 2078-2079.

3. DePristo MA, Banks E, Poplin R, Garimella KV, Maguire JR, et al. (2011) A framework for variation discovery and genotyping using next-generation DNA sequencing data. Nat Genet 43: 491-498.

4. McKenna A, Hanna M, Banks E, Sivachenko A, Cibulskis K, et al. (2010) The Genome Analysis Toolkit: a MapReduce framework for analyzing next-generation DNA sequencing data. Genome Res 20: 1297-1303.

5. Wysoker A, Tibbetts K, Fennell T (2011) Picard-Tools 1.5.3. Availble: <http://sourceforge.net/projects/picard/files/picard-tools/>. Accessed 2013 Aug. 25.

6. Challis D, Yu J, Evani US, Jackson AR, Paithankar S, et al. (2012) An integrative variant analysis suite for whole exome next-generation sequencing data. BMC Bioinformatics 13: 8.

7. Abecasis Lab (2010) Abecasis Lab GLF Tools. Available: <http://www.sph.umich.edu/csg/abecasis/glfTools/>. Accessed 2013 Aug. 25.

8. Danecek P, Auton A, Abecasis G, Albers CA, Banks E, et al. (2011) The variant call format and VCFtools. Bioinformatics 27: 2156-2158.

9. Wang K, Li M, Hakonarson H (2010) ANNOVAR: functional annotation of genetic variants from high-throughput sequencing data. Nucleic Acids Research 38: e164.

10. Abecasis GR, Altshuler D, Boehnke M, Daly M, McCarthy M, et al. (2012) Exome Chip Design.Available: <http://genome.sph.umich.edu/wiki/Exome_Chip_Design>. Accessed 2013 Aug. 25.

11. Homer N (2012) DWGSIM.Available: <http://sourceforge.net/apps/mediawiki/dnaa/index.php?title=Whole_Genome_Simulation>. Accessed 2013 Aug. 25.

12. Li H (2012) wgsim. Available: <https://github.com/lh3/wgsim>. Accessed 2013 Aug. 25.

13. CodonCode Corporation (2013) CodonCode Aligner. Available: <http://www.codoncode.com/aligner/>. Accessed 2013 Aug. 25.

**Figure Legends**

Figure S1. Raw variants from single-sample callings. a. Number of raw SNPs. b. Number of raw indels. c. Ti/Tv ratio in raw SNPs.

Figure S2. Filtered variants from single-sample callings. a. Number of filtered SNPs. b. Number of filtered indels. c. Ti/Tv ratio in filtered SNPs.

Figure S3. Variants from multiple-sample callings. a. Number of raw SNPs. b. Number of raw indels. c. Number of filtered SNPs. d. Number of filtered indels.

Figure S4. Validation of single-sample calling variants using exome array data. a. Number of true positive genotypes. b. Number of false positive genotypes. c. PPV, i.e., rediscovery rate. d. Sensitivity. e. Specificity.

Figure S5. Validation of multiple-sample calling variants using exome array data. a. Number of true positive genotypes. b. Number of false positive genotypes. c. PPV, i.e., rediscovery rate. d. Sensitivity. e. Specificity.
